# Insulin-like growth factor 2 reverses memory and synaptic deficits in APP transgenic mice

**DOI:** 10.15252/emmm.201404228

**Published:** 2014-08-07

**Authors:** Maria Pascual-Lucas, Silvia Viana da Silva, Marianna Di Scala, Carolina Garcia-Barroso, Gloria González-Aseguinolaza, Christophe Mulle, Cristina M Alberini, Mar Cuadrado-Tejedor, Ana Garcia-Osta

**Affiliations:** 1 Neurosciences Division, Center for Applied Medical Research, CIMA, University of NavarraPamplona, Spain; 2 Interdisciplinary Institute for Neuroscience, Université of Bordeaux, CNRS UMR 5297Bordeaux, France; 3 Gene Therapy and Hepatology Division, Center for Applied Medical Research, CIMA, University of NavarraPamplona, Spain; 4 Center for Neural Science, New York UniversityNew York, NY, USA; 5 Department of Anatomy, School of Medicine, University of NavarraPamplona, Spain

**Keywords:** Alzheimer's disease, IGF1, IGF2, IGF2R, synaptic plasticity

## Abstract

Insulin-like growth factor 2 (IGF2) was recently found to play a critical role in memory consolidation in rats and mice, and hippocampal or systemic administration of recombinant IGF2 enhances memory. Here, using a gene therapy-based approach with adeno-associated virus (AAV), we show that IGF2 overexpression in the hippocampus of aged wild-type mice enhances memory and promotes dendritic spine formation. Furthermore, we report that IGF2 expression decreases in the hippocampus of patients with Alzheimer's disease, and this leads us to hypothesize that increased IGF2 levels may be beneficial for treating the disease. Thus, we used the AAV system to deliver IGF2 or IGF1 into the hippocampus of the APP mouse model Tg2576 and demonstrate that IGF2 and insulin-like growth factor 1 (IGF1) rescue behavioural deficits, promote dendritic spine formation and restore normal hippocampal excitatory synaptic transmission. The brains of Tg2576 mice that overexpress IGF2 but not IGF1 also show a significant reduction in amyloid levels. This reduction probably occurs through an interaction with the IGF2 receptor (IGF2R). Hence, IGF2 and, to a lesser extent, IGF1 may be effective treatments for Alzheimer's disease.

## Introduction

Alzheimer's disease (AD) is the most common form of dementia in the elderly. There is currently no efficacious treatment for the disease. AD is characterized by β-amyloid (Aβ) accumulation (senile plaques), abnormal phosphorylation and aggregation of the microtubule-associated protein tau, synaptic dysfunction and neuronal death (Selkoe, 2011). Furthermore, several studies show that the accumulation of soluble forms of Aβ correlates with synapse dysfunction and loss (Shankar*et al*, 2007), which appears to occur relatively early in the disease, prior to cell death. Hence, agents that reverse synapse impairments and Aβ accumulation represent promising therapeutic candidates for the treatment and/or prevention of AD.

Recent studies in rodents have provided evidence of an important role for hippocampal insulin-like growth factor 2 (IGF2) in brain plasticity and learning and memory (Chen*et al*, 2011; Schmeisser*et al*, 2012). Moreover, recombinant IGF2 injected into the hippocampus significantly enhances memory retention and persistence (Chen*et al*, 2011). Systemic treatments with IGF2 significantly enhance hippocampal-cortical-dependent memories, including those known to be impaired in rodent models of AD (Stern*et al*, 2014). This would suggest that IGF2 is an interesting candidate for the treatment of cognitive impairment in AD. Furthermore, the formation and maturation of synapses in the hippocampus, a mechanism that is impaired in AD (Jacobsen*et al*, 2006; Smith*et al*, 2009; Ricobaraza*et al*, 2012) and depends on IGF2/IGF2R signalling, may underlie the IGF2-dependent memory enhancement effect (Schmeisser*et al*, 2012).

Unlike IGF2, insulin-like growth factor 1 (IGF1) has previously been researched as a potential treatment for AD. Studies have demonstrated that increasing levels of circulating IGF1 lowers amyloid β (Aβ) levels in the brain (Carro*et al*, 2002). This effect may be the result of the reduced serum-to-cerebrospinal fluid (CSF) traffic of IGF1 reported in both patients with AD (Johansson*et al*, 2013) and AD mouse models (Trueba-Saiz*et al*, 2013). However, other authors have demonstrated that reducing IGF1 receptor signalling protects against Aβ toxicity in different AD models (Cohen*et al*, 2009; Freude*et al*, 2009).

Based on current knowledge of the effects of IGF2 on synaptogenesis, brain plasticity and memory, we hypothesized that increasing IGF2 expression in the hippocampus may attenuate the memory deficits and synaptic dysfunction that occur in AD. We therefore analysed the expression of IGF2 in AD brains and employed an adeno-associated viral (AAV) vector gene-delivery system to express IGF2 in the hippocampus of the transgenic mouse model Tg2576 to test their effect on the behavioural, cellular and synaptic phenotypes typical of AD. The effects were compared to a group of animals injected with AAV expressing IGF1, since no memory enhancement effect has been attributed to this growth factor (Chen*et al*, 2011).

## Results

### AAV8-mediated expression of IGF2 reverses both memory and dendritic spine density impairments in aged wild-type mice

**Figure 1:**
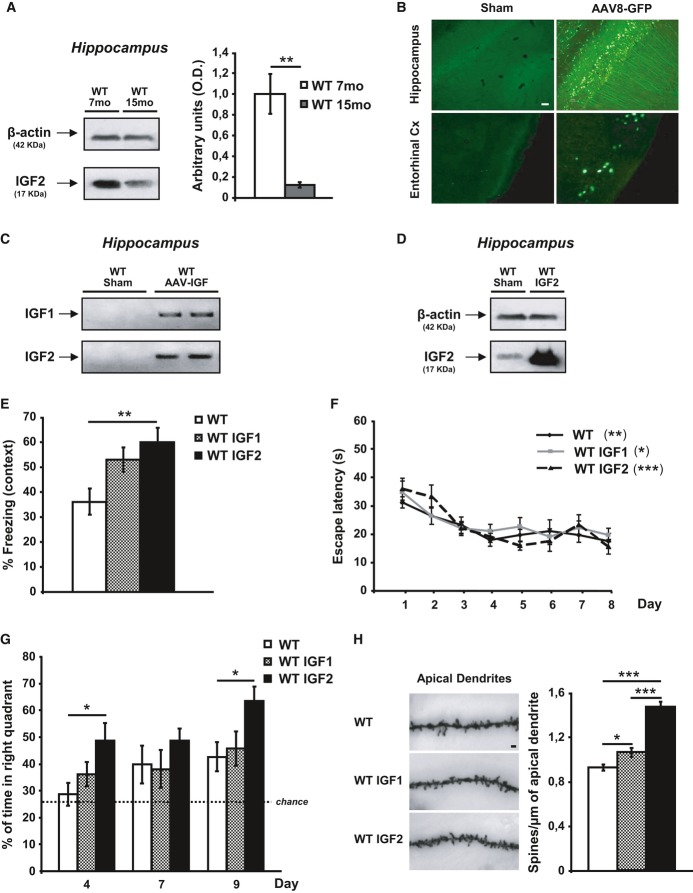
AAV8-mediated expression of IGF2 reverses both memory and dendritic spine density impairments in aged wild-type (WT) mice Quantitative Western blot analysis of hippocampal extracts that reveal a decrease in IGF2 in the hippocampus of aged (15-month-old) WT mice compared to 7-month-old WT animals. Data are expressed as arbitrary units (mean ± SEM) with respect to the WT 7-month-old mice (unpaired two-tailed Student's*t*-test,*n* = 4, **P = 0.01).Two months after the intrahippocampal administration of AAV8-GFP, GFP was detected in the pyramidal cells of CA1 and in neurons of the entorhinal cortex (Cx). No signal was detected in sham-injected animals. Scale bar = 10 μm.Gel analysis of PCR products obtained with the primers designed to determine the presence of AAV-mediated expression of IGF1 or IGF2 (WT AAV-IGF) in the hippocampus of WT mice. No band was detected in the sham-injected mice (WT sham).Western blot analysis of hippocampal extracts that reveal the overexpression of the IGF2 protein in the AAV-IGF2-injected mice compared to the sham-injected animals.Contextual freezing responses of aged WT mice 2 months after AAV-IGF2 injection (WT IGF2) showed a significant enhancement of memory retention measured as % freezing compared to the sham-injected mice (WT). In this, and all subsequent figures, results are expressed as mean ± SEM (Kruskal–Wallis followed by Mann–Whitney*U*-test,*n* = 10–12, **P = 0.0074).Escape latency to the hidden platform in the Morris water maze test for aged WT, WT IGF1 and WT IGF2 mice. Latency to reach the platform decreased in every group as the training sessions progressed (non-parametric Friedman test,*n* = 10–12, **P = 0.0029 WT, *P = 0.05 WT IGF1, ***P = 0.00026 WT IGF2). No differences were observed among the groups.Percentage of time spent searching for the target quadrant in the probe test (on days 4, 7 and 9). On days 4 and 9, WT IGF2 mice showed significant improvement in memory retention, measured as % of time spent in the right quadrant, compared to WT mice (one-way ANOVA followed by Scheffe's*post hoc* test,*n* = 10–12, *P = 0.043 day 4, *P = 0.050 day 9).Representative images of Golgi-impregnated apical dendrites of CA1 hippocampal pyramidal neurons. Scale bar = 10 μm (left panel). Quantification of overall spine density in 20-month-old WT mice (right panel). Spine density significantly increased in WT IGF1 and WT IGF2 mice compared to sham WT mice (Kruskal–Wallis followed by Mann–Whitney*U*-test,*n* = 36 neurons, *P = 0.025 WT versus WT IGF1, ***P = 1.03E-11 WT versus WT IGF2. The increase was greater for WT IGF2 mice, where spine density significantly increased compared to WT IGF1 (***P = 4.70E-08).

Since IGF2 has a role in memory processes, we studied whether age-related memory loss may be associated with changes in hippocampal IGF2 levels. By using quantitative Western blot analysis, the effect of age on endogenous IGF2 levels was measured in the hippocampus of 15-month-old versus 7-month-old wild-type (WT) animals. Interestingly, as depicted in Fig1A, the levels of endogenous IGF2 were significantly reduced with age in mice hippocampi. Next, we analysed whether restoring IGF2 levels could be effective in reversing age-related memory deficits. To achieve this, we studied whether the overexpression of hippocampal IGF2 could enhance memory in aged WT mice compared to the overexpression of IGF1. AAV vectors are widely used for gene transfer into the central nervous system (CNS), since they induce efficient and long-term transduction of non-dividing cells with no toxicity and a low immune reaction (Burger*et al*, 2005; Mandel*et al*, 2006). We chose AAV8 serotype, an efficient serotype that is non-toxic to neurons, to express IGF2 and IGF1 into the hippocampus (Cearley & Wolfe, 2006; Klein*et al*, 2006; Malik*et al*, 2012). AAV vectors encoding green fluorescent protein (GFP), IGF2 or IGF1 were developed as described in the Materials and Methods section and bilaterally injected into the hippocampus of 18-month-old WT mice. Two months later, AAV-GFP mice were euthanized to confirm the transduction efficiency of the AAV serotype in the hippocampus (Supplementary Fig S1). Long-term neuronal expression of GFP in the cell bodies of pyramidal CA1 neurons in the dorsal hippocampus and the neuropil was detected (Fig1B). Hippocampal neurons interact extensively with neurons in the entorhinal cortex (EC), a brain area that shows early signs of AD pathology and abundant amyloid plaques (Gomez-Isla*et al*, 1996). The expression of growth factors in the EC could contribute to their therapeutic potential. Interestingly, we found that some neurons in the EC of injected mice expressed GFP, which would indicate that the AAV delivered into the hippocampus infected and transduced cells in the EC. No signal was detected in non-infected mice (Fig1B).

To verify the viral expression of IGFs in the hippocampus of AAV-IGF-injected mice, PCRs were performed on the IGFs 3 months after the injections and compared to similar PCRs conducted on sham-injected mice. We performed conventional PCR using a 5′ primer designed against the 5′ end of the corresponding IGF cDNA and a 3′ primer aligned to the 3′ UTR-polyadenylation signal sequence of the recombinant IGF, which differs from that of the naturally expressed IGF mRNA (see Materials and Methods). As shown in Fig1C, AAV-IGF-injected mice had an amplified band that corresponded to the IGF expression in the hippocampus of injected mice (lanes 3 and 4). No band was detected in sham-injected hippocampi (sham, lanes 1 and 2).

Western blot analysis of hippocampal protein extracts obtained from WT mice (sham-injected) or injected with AAV-IGF2 and euthanized 3 months later revealed that hippocampal AAV-IGF2 administration resulted in a marked accumulation of IGF2 compared to sham-injected animals, and this lasted up to 3 months (Fig1D). Unfortunately, the IGF1 levels could not be determined by Western blot analysis, since no specific and sensitive antibody was available.

The memory capacity of aged AAV-IGF-treated mice was assessed using the fear-conditioning paradigm and the Morris water maze (MWM) test. The effects of AAV-mediated IGF1 and IGF2 expression on fear learning were tested in 20-month-old WT mice (2 months after the AAV injection). Animals were subjected to the milder single CS-US pairing protocol, which made it possible to detect subtler learning deficits. Freezing responses were significantly enhanced by AAV-IGF2 compared to sham mice. AAV-IGF1-treated mice showed weaker memory enhancement (Fig1E). These results demonstrate that IGF2 treatment reverses age-related defects in associative learning, which would suggest that synaptic function is enhanced.

The effect of IGF1 and/or IGF2 on MWM learning and spatial memory was also assessed in the mice. No significant differences were observed among the groups during the visible-platform training phase, which would indicate that the animals' ability to perform the task was similar. The groups did not differ in their swim speeds. No significant differences were observed among the groups during the invisible-platform training phase, and the animals were able to find the platform location (Fig1F). Interestingly, AAV-IGF2-treated mice showed significantly better memory retention in the retention phase on days 4 and 9 when compared to sham mice, whereas AAV-IGF1-treated mice behaved in a similar way to the control group (Fig1G). Interestingly, when compared to younger animals, AAV-IGF2-treated mice behaved in a similar way to 7-month-old WT animals during the probe test on days 4 and 7 (achieving 50% of time in the right quadrant) and performed even better in the probe test on day 9 (62 versus 50%, see Supplementary Fig S2).

Taken together, these results confirm that IGF2, and not IGF1, reverses age-related memory deficits in hippocampal-dependent learning tasks. The reposition of hippocampal IGF2 (down-regulated during aging, Fig1D) may underlie this memory enhancement observed in aged mice.

Dendritic spine loss is associated with the normal aging process, so we examined spine density using the Golgi-Cox staining method (Fig1H) to test whether the IGF2-induced behavioural recovery correlated with structural changes in dendritic spine density. The subregion CA1 was chosen for dendritic spine analysis since it has been shown as critical for contextual learning (Rampon*et al*, 2009). AAV-mediated expression of IGF2 significantly increased the apical dendritic spine density of CA1 pyramidal neurons. In the case of AAV-IGF1-injected mice, an increase in dendritic spine density was also detected, but the effect was significantly lower than in AAV-IGF2-injected mice (Fig1H). Here, we demonstrate that we developed an experimental memory-enhancing gene-therapy system through which functional IGF can be transferred into the mouse hippocampus with a single injection.

### IGF2 expression is reduced in the hippocampus of patients with AD and in Tg2576 mice

Previous studies have reported controversial results concerning the levels of IGF2 mRNA transcripts in the AD hippocampus compared to controls (Stein & Johnson, 2002; Stein*et al*, 2004; Steen*et al*, 2005). Here, we determined the IGF2 protein expression levels using Western blot analysis with an IGF2 antiserum (ab9574, Abcam, Cambridge, UK). We analysed IGF2 levels in human AD brain samples (EC and hippocampus), in Tg2576 mice hippocampi and in hippocampal neuronal cultures of Tg2576 mice or hippocampal neuronal cultures of WT mice treated with conditioned medium (CM) containing high concentrations of Aβ peptide, a procedure that induces a reduction in synaptic density similar to that found in AD (DaRocha-Souto*et al*, 2012).

**Figure 2:**
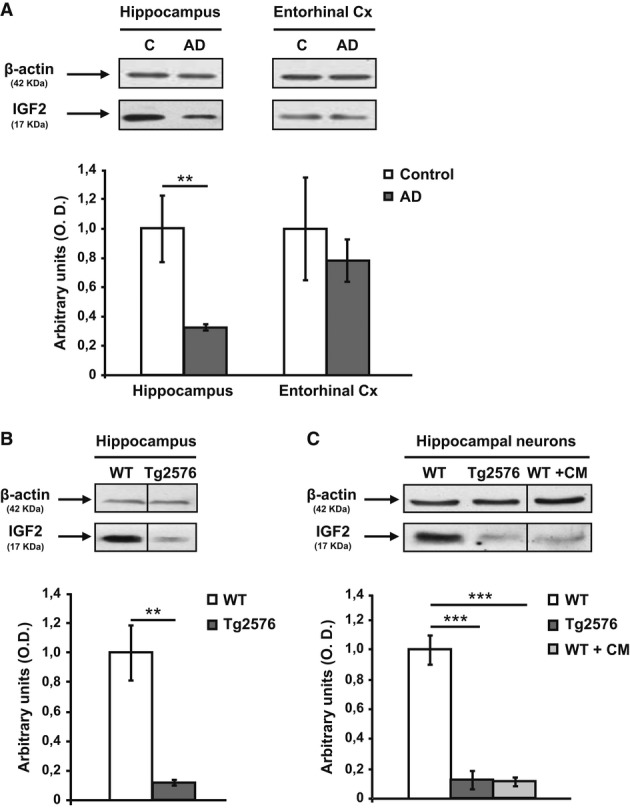
IGF2 protein levels are down-regulated in hippocampal samples of patients with AD and Tg2576 mice, and in Tg2576 neuronal culture Quantification of the relative expression of IGF2 protein by Western blot analysis and normalization to β-actin for protein load in: Hippocampal (*n* = 8) and entorhinal cortex (Cx,*n* = 6) from patients with AD compared to corresponding non-demented controls (*n* = 5 and*n* = 6 respectively). In this, and all subsequent figures, data are expressed as arbitrary units (mean ± SEM) with respect to their respective controls (unpaired two-tailed Student's*t*-test,*n* = 5–8, **P = 0.007).Seven-month-old Tg2576 mice hippocampus compared to age-matched WT mice (unpaired two-tailed Student's*t*-test,*n* = 4, **P = 0.010).Neuronal cultures of Tg2576 mice and neuronal cultures of WT mice exposed to conditioned medium (CM) obtained from Tg2576 primary neurons for 24 h compared to their respective control (one-way ANOVA followed by Scheffe's*post hoc* test,*n* = 5–6, ***P = 0.0006 WT versus Tg2576, ***P = 0.0003 WT versus WT + CM). Source data are available online for this figure.

Quantitative Western blot analyses showed a significant reduction in IGF2 in hippocampal samples from patients with AD (*n* = 8) (Braak stage V-VI) compared to non-AD control subjects (*n* = 5) of a similar age and sex (Fig2A). The dramatic decrease in IGF2 expression levels observed in the hippocampus of AD cases was not found in the EC of AD samples (*n* = 6 per group; Fig2A).

Next, IGF2 levels were determined in hippocampal extracts obtained from 7-month-old Tg2576 mice compared to age-matched WT mice. A significant decrease in IGF2 levels was found in the transgenic animals compared to age-matched WT mice (Fig2B). Similar results were also found in the hippocampal cultures of Tg2576 mice and in the WT hippocampal neuronal culture treated with Aβ (CM) (Fig2C) compared to those of WT mice, which would suggest a possible link between hippocampal Aβ accumulation and lower IGF2 concentration.

### AAV8-mediated expression of IGF1 and IGF2 in the AD mouse brain

Our results showed that IGF2 expression decreases in the hippocampus of patients with AD and in the Tg2576 mouse model of AD. This led us to hypothesize that IGF2 administration could improve AD symptoms. Thus, we used our AAV-mediated gene-transfer system (AAV-IGF2) as a therapeutic strategy for AD, where the transduction of IGF2 into the brain appears to be necessary.

**Figure 3:**
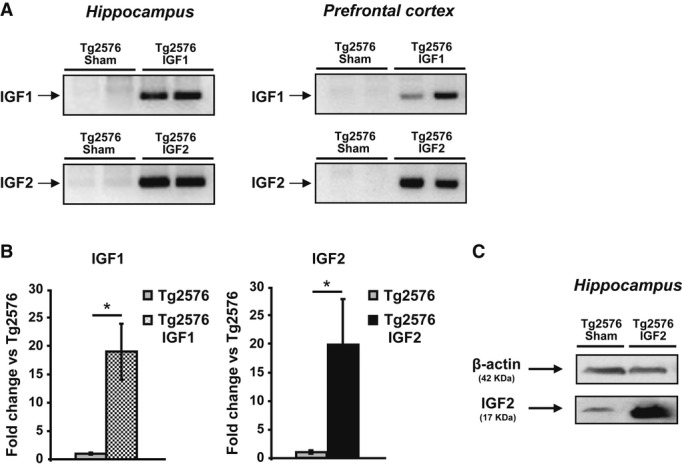
AAV-mediated expression of IGF1 and IGF2 in Tg2576 mouse brain Gel analysis of PCR products to determine the presence of AAV-mediated IGF1 and IGF2 expression. AAV-IGF-injected Tg2576 mice showed a PCR product corresponding to IGF expression in the hippocampus and the prefrontal cortex. No band was detected in the sham-injected mice (Tg2576 sham).Quantification of the relative expression of IGF transcripts by real-time PCR analysis using primers designed against the corresponding cDNAs of the murine IGFs. AAV-IGF-injected Tg2576 mice showed a robust increase in the corresponding IGF compared to their respective controls (Tg2576 sham). Data are expressed as the fold change (mean ± SEM) with respect to the controls (unpaired two-tailed Student's*t*-test,*n* = 5–6, *P = 0.036 Tg2576 IGF1, *P = 0.050 Tg2576 IGF2).Western blot analysis of hippocampal extracts revealing the overexpression of IGF2 protein in the AAV-IGF2-injected mice (Tg2576 IGF2) compared to Tg2576 sham. Source data are available online for this figure.

To determine the temporal profile of AAV-IGF expression in the hippocampus of Tg2576 mice, PCRs using primers for selective detection of the recombinant IGFs but not the endogenous ones (as described above) were carried out 4 and 8 months after the injections and compared to PCRs conducted on sham Tg2576 mice. As shown in Fig3A, AAV-IGF-injected mice had an amplified band that corresponded to the expression of AAV-IGF in the hippocampus of the injected mice (lanes 3 and 4). No band was detected in the hippocampi of sham-injected Tg2576 mice (sham, lanes 1 and 2).

Hippocampal neurons interact extensively with neurons in the EC and the prefrontal cortex (Braak*et al*, 1996). As also suggested by our viral expression described above (Fig1A), we tested whether the AAV-IGF tropism for connected brain regions led to IGF expression in the prefrontal cortex (Fig3A). The expression of growth factors in the other brain areas could contribute to their potential therapeutic effect. We detected PCR product amplification, which corresponded to AAV-IGF expression in the prefrontal cortex of the AAV-IGF-injected mice (lanes 3, 4), whereas no bands were detected in the sham-injected mice (sham: lanes 1 and 2). These results suggest that the AAV injected into the hippocampus also partially reaches and transduces cells in regions other than the hippocampus, such as the prefrontal cortex, and therefore may also elicit a protective effect there.

Furthermore, we performed quantitative real-time PCR using specific primers designed against the corresponding cDNA of the murine IGFs (to detect the recombinant and the endogenous IGFs). As depicted in Fig3B, AAV-IGF-injected mice showed a robust increase in the expression of the corresponding IGF in the hippocampus (Tg2576 IGF1 and Tg2576 IGF2) compared to sham-injected Tg2576 mice (Tg2576 sham).

The mRNA increase detected by PCR was confirmed in subsequent experiments that detected IGF protein levels. Western blot analysis of hippocampal protein extracts obtained from Tg2576 mice that were sham injected or injected with AAV-IGF2 and euthanized 8 months after the injections revealed that AAV-IGF2 injection produced a significant accumulation of IGF2 compared to sham injection, thus demonstrating the long-term expression of the recombinant protein for up to 8 months (Fig3C).

### IGF2 reverses both memory and dendritic spine density impairments in aged Tg2576 mice

**Figure 4:**
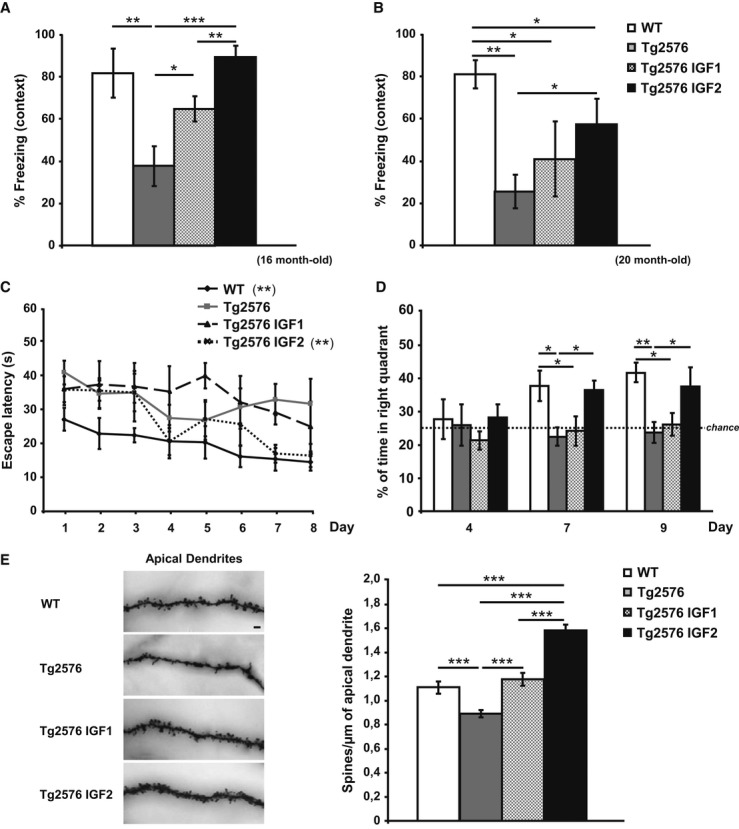
IGF2 reverses memory deficits and restores spine density in 16- and 20-month-old Tg2576 mice Contextual freezing responses of 16-month-old Tg2576 mice were impaired when compared to controls (WT), and significantly improved 4 months after the AAV-IGF1 (Tg2576 IGF1) and AAV-IGF2 (Tg2576 IGF2) injections. In this, and all subsequent figures, the results are expressed as mean ± SEM (Kruskal–Wallis followed by Mann–Whitney*U*-test,*n* = 10–12, **P = 0.008 WT versus Tg2576, *P = 0.041 Tg2576 versus Tg2576 IGF1, ***P = 0.0002 Tg2576 versus Tg2576 IGF2, **P = 0.0029 Tg2576 IGF1 versus Tg2576 IGF2).Memory impairments in Tg2576 mice progressed with age, and at 20 months, only Tg2576 IGF2 mice showed a significant improvement in memory retention, measured as % contextual freezing, compared to sham-injected Tg2576 mice (Tg2576) (Kruskal–Wallis followed by Mann–Whitney*U*-test,*n* = 10–12, **P = 0.0014 WT versus Tg2576, *P = 0.037 WT versus Tg2576 IGF1, *P = 0.039 WT versus Tg2576 IGF2, *P = 0.050 Tg2576 versus Tg2576 IGF2).Escape latency to the hidden platform in the Morris water maze test for WT, Tg2576, Tg2576 IGF1 and Tg2576 IGF2 mice. Latency to reach the platform decreased in WT and Tg2576 IGF2 mice as the training sessions progressed. No such change was observed in Tg2576 IGF1 or Tg2576 mice (non-parametric Friedman test,*n* = 10–12, **P = 0.002 WT, **P = 0.005 Tg2576 IGF2).Percentage of time spent searching for the target quadrant in the probe test (days 4, 7 and 9). Tg2576 mice performed significantly more poorly than WT mice. AAV-IGF2-injected groups performed similarly to WT mice on days 7 and 9 (one-way ANOVA followed by Scheffe's*post hoc* test,*n* = 10–12, *P = 0.045 WT versus Tg2576 day 7, *P = 0.05 WT versus Tg2576 IGF1 day 7, *P = 0.049 Tg2576 versus Tg2576 IGF2 day 7, **P = 0.007 WT versus Tg2576 day 9, *P = 0.044 WT versus Tg2576 IGF1 day 9, *P = 0.035 Tg2576 versus Tg2576 IGF2 day 9).Representative images of Golgi-impregnated apical dendrites of CA1 hippocampal pyramidal neurons. Scale bar = 10 μm (left panel). Quantification of overall spine density in 20-month-old Tg2576 mice (right panel). Significant reduction in spine density was detected in 20-month-old Tg2576 mice compared to WT mice (Kruskal–Wallis followed by Mann–Whitney*U*-test,*n* = 27–36 neurons, ***P = 0.0009 WT versus Tg2576). AAV-IGF1 treatment in Tg2576 mice (Tg2576 IGF1) completely reversed spine loss, which returned to littermate control levels (WT) (***P = 1.67E-05 Tg2576 versus Tg2576 IGF1). AAV-IGF2 treatment (Tg2576 IGF2) increased spine density to above control values (***P = 6.05E-08 Tg2576 IGF2 versus WT, ***P = 1.94E-12 Tg2576 IGF2 versus Tg2576, ***P = 2.86E-06 Tg2576 IGF2 versus Tg2576 IGF1).

The memory capacity of AAV-IGF-treated mice was assessed using two hippocampal-dependent tasks: contextual fear conditioning and the MWM. The effects of AAV-mediated IGF1 and IGF2 expression on contextual fear learning and memory were tested in Tg2576 transgenic mice at two different ages: 16 months old (4 months after the AAV injection) and 20 months old (8 months after the AAV injection). Compared to WT mice, and in line with previous studies (Ricobaraza*et al*, 2012), the Tg2576 mice at both ages exhibited severe disruption in freezing behaviour in the contextual fear-conditioning task conducted 24 h after training (Fig4A and B). However, AAV-IGF2 significantly rescued memory impairment at both 16 months (Fig4A) and 20 months old (Fig4B), whereas AAV-IGF1 showed only a significant effect at 16 months of age (Fig4A).

The effect of IGF1 and IGF2 on MWM learning and spatial memory was only assessed in 20-month-old mice (8 months after the AAV injection), since it has already been established that Tg2576 mice at this age present impairments in both acquisition and retention phases (Westerman*et al*, 2002). No significant differences were observed among the groups during the visible-platform training phase, which would indicate that the animals' ability to perform the task was similar. Furthermore, the groups did not differ in their swim speeds. The effect of IGF1 and/or IGF2 on spatial learning was assessed according to the variance of intra-group latency (over trials), which was analysed using the non-parametric Friedman test (see Materials and Methods). The analysis showed that, while the mean latency to reach the platform significantly decreased in WT mice and in AAV-IGF2-treated Tg2576 mice as the training sessions progressed, such a change was not observed in AAV-IGF1 or sham Tg2576 mice (Fig4C). On days 4, 7 and 9, the mice were subjected to a retention probe trial in which the platform was removed. No differences were observed among the groups on the first probe (day 4); however, on days 7 and 9, sham and AAV-IGF1-treated Tg2576 mice showed a significantly poorer performance when compared to WT littermates, whereas AAV-IGF2-treated Tg2576 mice behaved in a similar way to WT littermates (Fig4D). Taken together, these results indicate that IGF2 (Fig4A–D) reverses memory deficits in Tg2576 mice.

Given that impaired synaptic function may underlie memory deficits in AD, and that synapse dysfunction and dendritic spine loss develop in Tg2576 mice (Ricobaraza*et al*, 2012), we tested whether IGF2-induced behavioural recovery correlates with structural changes in dendritic spine density. To this end, we examined spine density using the Golgi-Cox staining method (Fig4E). A significant decrease in spine density of apical dendrites of CA1 pyramidal neurons was found in 20-month-old Tg2576 mice compared to WT mice, in line with previous findings (Ricobaraza*et al*, 2012). Compared to sham mice, AAV-mediated IGF1 or IGF2 expression significantly reversed the deficits in apical dendritic spine density of CA1 pyramidal neurons, with AAV-IGF1 causing the levels to return to control values and AAV-IGF2 causing an increase (Fig4E).

### Both IGF1 and IGF2 rescue hippocampal mEPSCs in Tg2576 mice

**Figure 5:**
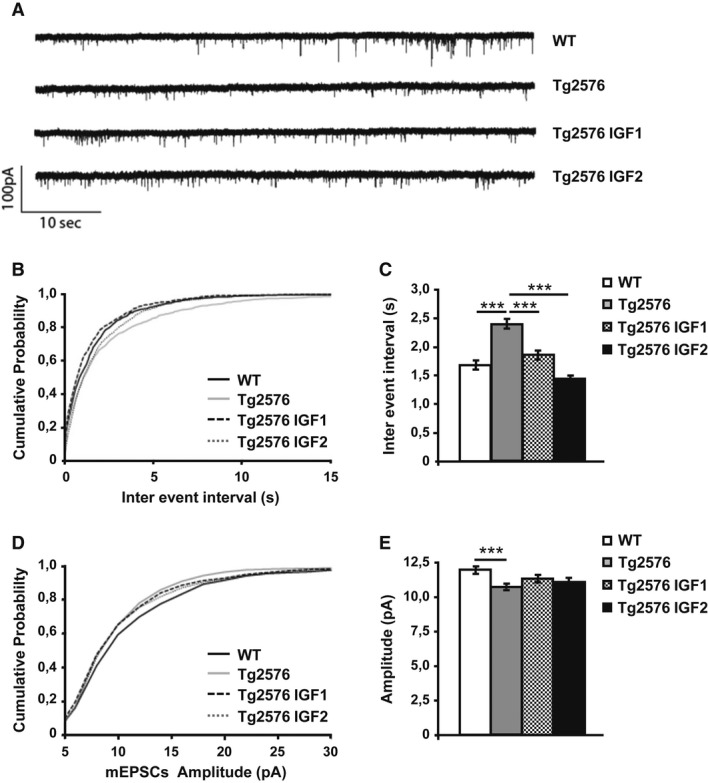
IGF1 and IGF2 treatment restores CA1 synaptic transmission to control levels Miniature EPSCs were recorded from CA1 pyramidal cells in 7-month-old animals 4 months after treatment. Cells were held at -70 mV in the presence of bicuculline (10 μM) and TTX (1 μM). Representative traces of spontaneous AMPA EPSCs were isolated from the first 15 min after whole cell configuration.Cumulative probability analysis of the distribution of inter-event intervals between mEPSCs showed a very significant difference between WT and Tg2576 (non-parametric Kolmogorov–Smirnov test,*n* = 10,*P* = 0.0002). Treatment with IGF1 and IGF2 rescued the frequency of mEPSCs to values that were no different from those of the control animals (*P* = 0.0129 Tg2576 versus Tg2576 IGF1,*P* < 0.0001 Tg2576 versus Tg2576 IGF2).Average mEPSC interval between the first 100 events of 10 cells per treatment (one-way ANOVA followed by Tukey's*post hoc* test,*n* = 10, ***P < 0.001).Cumulative probability analysis of mEPSC amplitude showed a difference between Tg2576 and WT (non-parametric Kolmogorov–Smirnov test,*n* = 10,*P* = 0.0003). The mEPSC amplitude of the treated groups was not rescued to the values of the WT animals (*P* = 0.3136 Tg2576 versus Tg2576 IGF1,*P* = 0.4658 Tg2576 versus Tg2576 IGF2).Average amplitude of the first 100 events of 10 cells per treatment (one-way ANOVA followed by Tukey's*post hoc* test,*n* = 10, ***P < 0.001).

Given that both IGF1 and IGF2 expression rescued the dendritic spine density deficit in Tg2576 mice, we tested the effects of IGFs on synaptic transmission of CA1 and CA3 pyramidal cells. AAV-IGFs were bilaterally injected into the hippocampus of 4-month-old Tg2576 mice, and 3 months later, the mEPSCs in whole-cell patch-clamp mode from CA1 and CA3 pyramidal cells were recorded (7-month-old Tg2576 mice). It has been described in literature that at this age, there is already reduction in spine number in the hippocampus (Ricobaraza*et al*, 2012) and decrease in IGF2 levels (Fig2B). In the presence of bicuculline (10 μM) and TTX (1 mM), mEPSC frequency was assessed by measuring inter-event intervals (IEI). We found that mEPSC frequency was markedly decreased in the pyramidal cells of both CA1 (Fig5B and C) and CA3 (Supplementary Fig S3B and C) of Tg2576 mice, as indicated by the increase in inter-event intervals (IEI). IGF1 or IGF2 expression fully reversed the deficit in mEPSC frequency. This result is consistent with the spine density recovery observed in AAV-IGF1 and AAV-IGF2 mice (Fig4E).

The average mEPSC amplitude decreased in the pyramidal cells of both CA1 (Fig5D and E) and CA3 of Tg2576 mice (Supplementary Fig S3D and E). Although the difference was highly significant, the reduction was only in the order of 20%. This reduction in mEPSC amplitude can be explained by a decrease in the average density of postsynaptic AMPA receptors or alternatively by a change in their biophysical properties. Neither IGF1 nor IGF2 changed the mEPSC amplitude (Fig5D and E; Supplementary Fig S3D and E).

### IGF2 but not IGF1 reduces brain Aβ levels and amyloid plaques in 20-month-old Tg2576 mice

**Figure 6:**
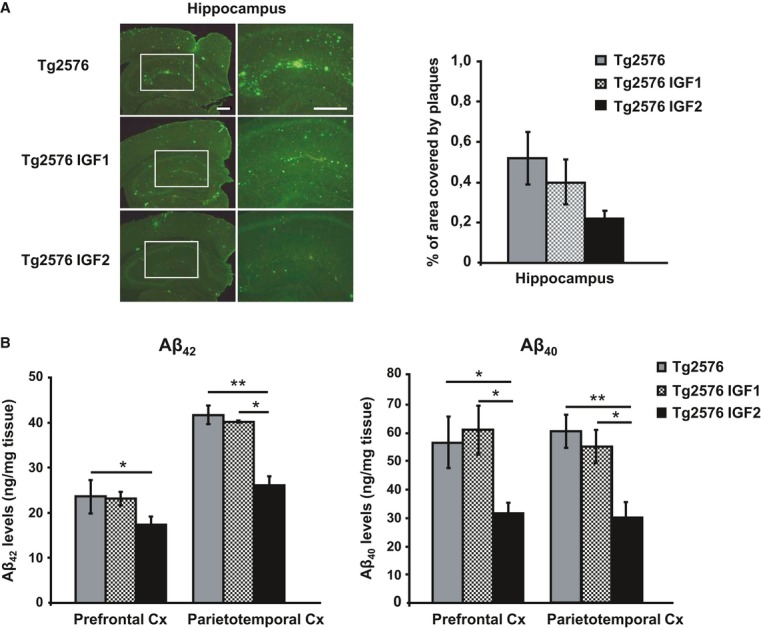
IGF2 reduces amyloid burden in 20-month-old Tg2576 mice Representative hippocampal sections of sham- (Tg2576), AAV-IGF1- (Tg2576 IGF1) and AAV-IGF2- (Tg2576 IGF2) treated Tg2576 mice are shown (left panel). Scale bar = 100 μm. Amyloid burden quantification (right panel). Multiple extracellular deposits stained with 6E10 antiserum were detected in Tg2576 and Tg2576 IGF1 mice. Amyloid burden is reduced in the hippocampus of Tg2576 IGF2 mice (*n* = 3–5); however, no significant differences were found.Aβ_42_ and Aβ_40_ concentration determined by ELISA in the prefrontal and parietotemporal cortices (Cx) of Tg2576 mice and Tg2576 IGF1 mice showed similar values. Interestingly, Tg2576 IGF2 mice exhibited a significant reduction in Aβ_42_ (one-way ANOVA followed by Scheffe's*post hoc* test,*n* = 7–8, *P = 0.050 Tg2576 versus Tg2576 IGF2 prefrontal Cx, **P = 0.007 Tg2576 versus Tg2576 IGF2 parietotemporal Cx, *P = 0.038 Tg2576 IGF1 versus Tg2576 IGF2 parietotemporal Cx) and Aβ_40_ (*P = 0.019 Tg2576 versus Tg2576 IGF2 prefrontal Cx, *P = 0.014 Tg2576 IGF1 versus Tg2576 IGF2 prefrontal Cx, **P = 0.003 Tg2576 versus Tg2576 IGF2 parietotemporal Cx, *P = 0.038 Tg2576 IGF1 versus Tg2576 IGF2 parietotemporal Cx) cortical levels. Data are the mean ± SEM.

Next, we examined the potential mechanisms involved in IGF-induced spine density and synaptic transmission recovery in the APP model. It is thought that synaptic impairments in AD are triggered by Aβ accumulation resulting from an imbalance between Aβ production and clearance (Hardy & Selkoe, 2002). Thus, amyloid pathology was analysed in the group of 20-month-old Tg2576 mice overexpressing IGF1 or IGF2 and compared to sham Tg2576 mice (8 months after injection). The amyloid burden analysis in the hippocampus was assessed by immunofluorescence using the monoclonal antibody 6E10, which recognizes the amino-terminal region of Aβ, and quantified using the analySIS image system (Gomez-Isla*et al*, 1996; Ribe*et al*, 2005). As depicted in Fig6A, a high density of amyloid plaques was found in the hippocampus of 20-month-old Tg2576 mice, in line with previous findings (Ribe*et al*, 2005). A marked reduction in the hippocampal amyloid burden was detected in AAV-IGF2 Tg2576 mice, whereas no change was observed in AAV-IGF1 mice compared to sham-injected Tg2576 mice (Fig6A).

Next, levels of Aβ_42_ and Aβ_40_ were determined by ELISA in the prefrontal cortex and in the parietotemporal cortex, regions that are extensively affected by amyloid burden in Tg2576 mice (Ribe*et al*, 2005). A significant reduction of 26 and 38% in Aβ_42_ levels, and 24 and 50% in Aβ_40_ levels, was observed in the prefrontal and the parietotemporal cortex, respectively, of AAV-IGF2-treated Tg2576 mice compared to sham-injected controls (Fig6B). No changes were found in AAV-IGF1-treated Tg2576 mice compared to sham-injected Tg2576 mice (Fig6B). To confirm the effect of AAV-IGF2 on the improvement in amyloid burden in cortical areas, the number of amyloid plaques was also quantified in the EC and motor cortex by immunofluorescence using the monoclonal antibody 6E10. A reduction in amyloid burden was found in AAV-IGF2-treated mice compared to sham-injected mice (Supplementary Fig S4).

Hence, hippocampal IGF2, but not IGF1, rescues amyloid burden typical of AD.

### IGF2/IGF2R facilitates Aβ clearance

**Figure 7:**
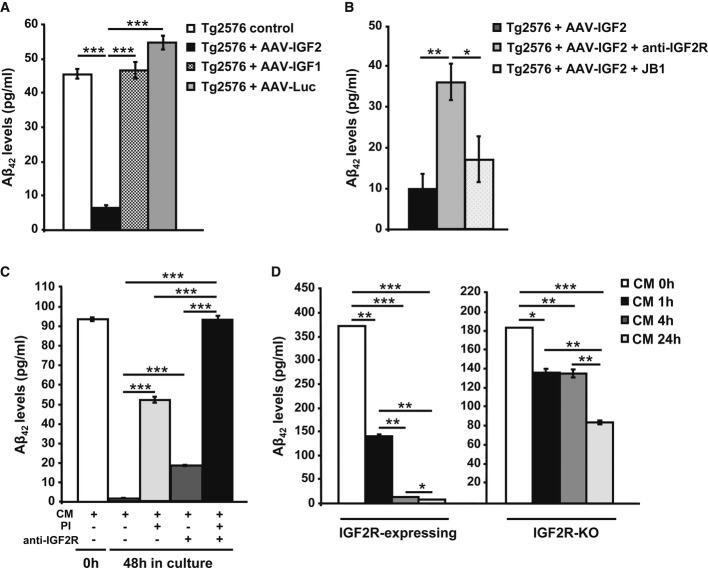
IGF2/IGF2R mediates Aβ_42_ clearance in neurons ELISA analysis to detect Aβ_42_ levels in the media of Tg2576 primary neuronal culture infected with AAV-IGF1, AAV-IGF2 or AAV-Luc. A significant depletion in extracellular Aβ_42_ levels was detected in the media of AAV-IGF2-infected neurons. In this, and all subsequent figures, data (mean ± SEM) are expressed in pg of Aβ_42_/ml of conditioned medium. Bars represent the analysis from three independent experiments (one-way ANOVA followed by Scheffe's*post hoc* test,*n* = 3–4, ***P = 3.12E-08 Tg2576 control versus Tg2576 + AAV-IGF2, ***P = 1.91E-08 Tg2576 + AAV-IGF2 versus Tg2576 + AAV-IGF1, ***P = 2.39E-09 Tg2576 + AAV-IGF2 versus Tg2576 + AAV-Luc).ELISA analysis to detect Aβ_42_ levels in the medium from Tg2576 primary neuronal cultures transfected with AAV-IGF2. Pre-incubation with anti-IGF2R antibody partially prevented Aβ_42_ clearance in the media of infected neurons. Bars represent the analysis from three independent experiments (one-way ANOVA followed by Scheffe's*post hoc* test,*n* = 3–4, **P = 0.009, *P = 0.036).ELISA analysis to detect Aβ_42_ levels in media obtained from WT primary neuronal cultures exposed to conditioned medium (CM) from Tg2576 neurons for 48 h. Full depletion in extracellular Aβ_42_ occurred after 48 h; this was prevented by pre-incubation with proteinase inhibitors (PI) and/or anti-IGF2R antibody (one-way ANOVA followed by Scheffe's*post hoc* test,*n* = 3–4, ***P = 2.65E-09 CM versus CM + PI, ***P = 3.56E-05 CM versus CM + anti-IGF2R, ***P = 1.26E-11 CM versus CM + PI + anti-IGF2R, ***P = 1.57E-08 CM + PI versus CM + PI + anti-IGF2R, ***P = 7.80E-11 CM + anti-IGF2R versus CM + PI + anti-IGF2R).Analysis by sandwich ELISA of Aβ_42_ levels at different time intervals (0, 1, 4 and 24 h) in the media of hepatic cell lines (BNL.CL.2 and HepA129; IGF2R-expressing and IGF2-KO, respectively) exposed to conditioned medium from Tg2576 primary neurons (CM). After 4 h of incubation, extracellular Aβ_42_ was completely eliminated from the IGF2R-expressing cell medium (one-way ANOVA followed by Scheffe's*post hoc* test,*n* = 3–4, **P = 0.002 CM 0 h versus CM 1 h, ***P = 1.99E-05 CM 0 h versus CM 4 h, ***P = 8.23E-06 CM 0 h versus CM 24 h, **P = 0.006 CM 1 h versus CM 4 h, **P = 0.006 CM 1 h versus CM 24 h, *P = 0.02 CM 4 h versus CM 24 h), but not from the IGF2R-KO cell medium, where Aβ_42_ was detected even after 24 h (*P = 0.011 CM 0 h versus CM 1 h, **P = 0.009 CM 0 h versus CM 4 h, ***P = 0.0001 CM 0 h versus CM 24 h, **P = 0.002 CM 1 h versus CM 24 h, **P = 0.0014 CM 4 h versus CM 24 h). Bars represent the analysis from three independent measurements.

To begin elucidating the mechanisms by which IGF2 improves amyloid pathology in Tg2576 mice,*in vitro* experiments were carried out. Tg2576 neuronal cultures (DIV 10) were infected with AAV-IGF2, AAV-IGF1 or AAV-luciferase (Luc). After 3 days, the Aβ_42_ levels in the cell media were determined using ELISA. A significant reduction in Aβ_42_ levels (Fig7A) was observed in the media of primary Tg2576 neurons infected with AAV-IGF2 compared to non-infected neurons (Tg2576 control). There was no significant difference in the Aβ_42_ levels in the media of Tg2576 neurons infected with AAV-IGF1 or AAV-Luc versus the control (Fig 7A).

IGF2 mediates its actions mainly through the IGF2R, but it also binds to the IGF1R with a lower affinity. To determine which receptor (IGF2R or IGF1R) mediates the effect of IGF2 on Aβ_42_ reduction, AAV-IGF2-infected neurons were pre-incubated with a blocking anti-IGF2R antibody (2 ng/μl) or the selective IGF1R antagonist JB1 (500 pg/μl) 24 h before media collection. The extracellular Aβ_42_ reduction was significantly blocked when neurons were exposed to anti-IGF2R antibody. No effect was observed with JB1 (Fig7B), which would indicate that IGF2R mediates the effect of IGF2 on Aβ_42_ load reduction, while IGF1R does not.

Next, we analysed whether the IGF2R-mediated reduction in Aβ_42_ levels results from an increased clearance of Aβ_42_. To this end, we used CM obtained from Tg2576 neuronal cultures (Tg2576-CM) as a natural source of Aβ_42_ to treat the WT neuronal culture. After 48 h, Aβ_42_ (measured by ELISA) was entirely depleted from the media (Fig7C, CM), which demonstrates that neurons degrade extracellular Aβ_42_. The degradation was partially blocked when cells were incubated for 48 h with Tg2576-CM containing a collection of proteinase inhibitors (PI) (Fig7C, CM + PI), which would suggest the involvement of other cell-mediated clearance processes in Aβ_42_ elimination. To determine whether IGF2R is involved in these processes, cells exposed to Tg2576-CM were also incubated with a blocking anti-IGF2R antibody (CM+anti-IGF2R). In these conditions, extracellular Aβ_42_ degradation was also partially blocked. Furthermore, when neurons exposed to Tg2576-CM were incubated with both PI and anti-IGF2R antibody (CM + PI + anti-IGF2R), Aβ_42_ degradation was entirely blocked (Fig7C). These results confirm that IGF2R plays an important role in Aβ_42_ clearance processes in neurons.

To verify that IGF2R plays a role in the clearance of Aβ_42_, we employed a murine hepatoma cell line (HepA129), which does not express IGF2R (IGF2R-KO). Cells were exposed to Tg2576-CM, and Aβ_42_ levels were analysed using ELISA at different time intervals after incubation (1, 4 and 24 h). As a control, mouse embryonic liver hepatocyte-like cells, BNL.CL.2, which express IGF2R (IGF2R-expressing), were used. Remarkably, after 4 h of incubation, extracellular Aβ_42_ was completely eliminated from the BNL.CL.2 cell media, but not from the HepA129 cell media, where Aβ_42_ was detected even after 24 h (Fig7D). These results support the hypothesis that IGF2R may act as an Aβ_42_ scavenger.

## Discussion

In this study, we used AAV to develop an experimental gene-therapy system for transferring functional IGF2 into the mouse hippocampus for a long time with a single injection. We observed for the first time that the levels of endogenous IGF2 were significantly decreased with age in the mice hippocampi. This observation further supports the potential involvement of IGF2 in age-dependent memory deterioration. Thus, we conducted*in vivo* experiments demonstrating that IGF2 enhances memory and induces synapse formation in the hippocampus of WT rodents. Furthermore, after observing that IGF2 levels decreased in the hippocampus of patients with AD and in the Tg2576 mouse model, we used AAV to deliver IGF2 and IGF1 into the hippocampus of Tg2576 mice to determine their potential therapeutic efficacy. We demonstrated that AAV-IGF2-injected Tg2576 mice performed significantly better in memory tests, showed increased spine density and excitatory synaptic transmission and presented significantly lower amyloid levels than sham Tg2576 mice. A partial recovery in both memory and synaptic transmission was also observed in AAV-IGF1-injected Tg2576 mice. Furthermore, IGF2 was identified for the first time as a regulator of amyloid levels in neuronal population, probably through the IGF2R. These findings suggest that IGF2 has potential therapeutic properties that could be beneficial for the treatment of AD.

IGF2 is mainly produced during foetal development. Although its levels drop in adulthood, they remain higher in the brain than other members of the insulin system. In line with previous findings (Rivera*et al*, 2005), we observed that IGF2 protein levels decline in the hippocampus of the AD brain; this led us to test the hypothesis that IGF2 is a potential treatment for AD in preclinical models. We demonstrated that IGF2 overexpression in the hippocampus of Tg2576 mice after the onset of severe AD improved hippocampal-dependent memory and synaptic deficits in these mice. In addition, when AAV-IGF2 was injected into the hippocampus of aged WT mice, the animals showed an improvement in memory performance. Memory impairments in Tg2576 mice and age-related memory loss are associated with spine loss in pyramidal CA1 neurons (Ricobaraza*et al*, 2012) and reduced mEPSC frequency in CA1 pyramidal neurons in Tg2576 mice as young as 3 months old (D'Amelio*et al*, 2011). We observed that IGF2-induced cognitive recovery in both the aged and AD model was accompanied by an increase in spine density and the rescue of synaptic functions through an increase in mEPSC frequency in the AD transgenic mouse model. This is consistent with a recent finding that IGF2-IGF2R signalling promotes synapse formation and spine maturation in the mouse brain (Schmeisser*et al*, 2012). These results are also in line with evidence that IGF2 facilitates memory consolidation and long-term hippocampal potentiation in rats (Chen*et al*, 2011). Furthermore, IGF2 may affect neurogenesis (Bracko*et al*, 2012) and, through this mechanism, contribute to hippocampal-dependent spatial learning and memory, which has been linked to neurogenesis (Bracko*et al*, 2012; Ouchi*et al*, 2013). In keeping with this, the hippocampal expression of IGF2 was found to rescue adult neurogenesis and improve spatial working memory deficits in the Dgcr8 (DiGeorge syndrome chromosomal region 8) deficient mouse model of schizophrenia (Ouchi*et al*, 2013). Furthermore, an increase in IGF2 contributes to the improvement in cognitive dysfunction by several pharmacological treatments, such as galantamine, choline or dicholine succinate (Cline*et al*, 2012; Kita*et al*, 2013) (Napoli*et al*, 2008). Hence, our results contribute to the body of evidence accumulated in recent years that IGF2 plays an important role in learning and memory formation, and demonstrate that by restoring dendritic spine density, IGF2 may significantly promote the recovery of memory impairments.

Hippocampal IGF1 overexpression in Tg2576 mice also resulted in the recovery of hippocampal spine density and restoration of synaptic transmission. However, when memory was assessed in 20-month-old mice, Tg2576 AAV-IGF1-injected mice failed to learn the tasks and were thus found to have significant impairments, whereas Tg2576 AAV-IGF2-injected mice had learning abilities that were similar to those of non-transgenic mice. Hence, despite the fact that IGF1 has some effect, our results suggest that IGF2 is far more effective. This was confirmed for age-related memory loss, since AAV-IGF2-injected mice performed significantly better than the AAV-IGF1-treated group (Fig1E and F). Furthermore, the results are consistent with the fact that IGF2R is the receptor that mediates the effect of IGF2 on synaptogenesis (Schmeisser*et al*, 2012) and memory formation (Chen*et al*, 2011). In addition, IGF2R exhibits a much lower binding affinity for IGF1 (El-Shewy & Luttrell, 2009). Although a possible role for IGF1 upon activation of IGF1R via Akt signalling cannot be ruled out, further studies are required to shed light on the role of IGF1 on memory function and synapse formation.

Crucially, we showed that IGF2 overexpression in the hippocampus leads to a significant reduction in Aβ content in the brains of Tg2576 mice. However, this effect was not observed in AAV-IGF1-injected mice. These results do not correlate with those of previous studies that demonstrated that the systemic administration of IGF1 promotes Aβ clearance from the brain (Carro*et al*, 2002, 2006). The authors suggest that circulating IGF1 increases Aβ carrier proteins and thus favours Aβ clearance from the brain. It is important to note that hippocampal IGF1 overexpression may trigger a different effect on amyloid pathology than increasing levels of circulating IGF1. In accordance with our data, there is a very recent study that addresses the role of IGF2 in the amelioration of amyloid-osis using a different AD mouse model (Mellott*et al*, 2014). Mellott*et al* found a decrease in amyloid deposits in APP/PS1 mice brain after 1 week of intraventricular infusion of human recombinant IGF2. They suggested that an enhance in cholinergic transmission and the synthesis of neuronal growth factors may be the mechanisms involved in IGF2-mediated Aβ clearance (Mellott*et al*, 2014).

In our study, in relation to the mechanism by which IGF2 improves Aβ pathology in Tg2576 mice, the results obtained in the*in vitro* assays suggest that the IGF2R appears to be involved in the protease-independent Aβ clearance mechanisms by neurons. The presence of the antagonistic anti-IGF2R antibody partially blocked Aβ clearance from the media of neuronal cultures expressing IGF2. An upregulation of IGF2R levels was observed in the hippocampus of AAV-IGF2-injected Tg2576 mice (Supplementary Fig S5). IGF2R is known to be involved in protein processing and clearance, primarily through lysosomal degradation, since IGF2R mediates lysosomal enzyme trafficking (El-Shewy & Luttrell, 2009). The extracellular fraction of IGF2R contains 15 homologous domains of 147 amino acids. The IGF2-binding domain has been mapped to domain 11, whereas M6P residues bind domains 3 and 9 (Braulke, 1999). Aβ has recently been shown to precisely bind IGF2R domain 11, which would suggest that IGF2R may act as an Aβ scavenger (Murakoshi*et al*, 2013). Taken together, these results demonstrate for the first time, to our knowledge, that IGF2/IGF2R is involved in the extracellular Aβ degradation mechanism, and suggest that IGF2R may be involved in the Aβ clearance observed in AAV-IGF2-injected Tg2576 mice.

Thus, IGF2 may represent an effective therapeutic target for the memory, synaptic loss and amyloid accumulation that accompany AD pathology.

## Materials and Methods

### Viral construction, production and purification

For construction of the recombinant viral genomes AAV-IGF1 and AAV-IGF2, IGF1 (GenBank accession number NM_010512.4) and IGF2 (GenBank accession number NM_001122737.1) cDNAs were amplified using gene specific primers where the 5′-primer contained a*XbaI* restriction site and the 3′-primer contained a*BglII* restriction site, and cDNA obtained from foetal hippocampus brain as template. The PCR products were subsequently cloned into pCR2.1 to obtain pCR2.1-IGF1 and pCR2.1-IGF2. The IGF1/IGF2 fragments were excised with*XbaI* and*BglII* and cloned into dsAAV-EF1α-eGFP (Applied Viromics, Fremont, CA) previously digested with*XBaI* and*BglII* to obtain the plasmids dsAAV-EF1α- IGF1 and dsAAV-EF1α-IGF2. This plasmid carries the AAV8 backbone with an intact 5′ terminal resolution site (trs) without the 3′trs analogous. Thus, the expression of IGF1 and IGF2 is under the transcriptional control of the constitutive and ubiquitous promoter of human elongation factor-1 (GenBank accession number J04617.1).

rAAV8 vectors were produced by polyethylenimine (PEI)-mediated cotransfection in HEK-293 cells as previously described (Paneda*et al*, 2009). For each production, a mixture of plasmids, 20 μg of pro-AAV plasmid and 55 μg pDP8.ape (PlasmidFactory GmbH & Co. KG, Bielefeld Germany), was transfected into 293 cells 15 cm plate using linear PEI 25 kDa (Polysciences, Warrington, PA) and harvested 72 h after transfection, and virus was released from the cells by three rounds of freeze-thawing. Crude lysate from all batches was then treated with Benzonase (50 U/ml crude lysate) for 1 h at 37°C and then kept at −80°C until purification. Purification of crude lysate was performed by iodixanol gradients according to the method of Zolotukhin*et al* (1999). The purified batches were concentrated and diafiltrated by cross-flow filtration (Spectrum Laboratories, Rancho Dominguez, CA) with a molecular mass cut-off of 400 kDa. The batches were then concentrated further by passage through Centricon tubes (YM-100; Millipore, Bedford, MA). After concentration, the viral batches were filtered (pore size, 0.22 mm) and stored at −80°C. Viral titres in terms of genome copies per millilitre were determined by Q-PCR, performed three times in triplicate at three different dilutions.

### Animals

A group of aged WT animals was used to test the effect of IGF2 and IGF1 on cognitive function. For this aim, male 18-month-old C57BL/6 mice were used. Animals were housed 4–5 per cage with*ad libitum* access to food and water and maintained in a temperature-controlled environment on a 12-h dark/light cycle. Next, as a model of AD, we used transgenic mice Tg2576 overexpressing human 695-aa isoform of amyloid precursor protein (hAPP) carrying the Swedish double mutation (APPswe: K670N, M671L) driven by a hamster prion promoter (Hsiao*et al*, 1996). The mice were on an inbred C57BL/6/SJL genetic background. Behavioural and biochemical studies were performed comparing Tg2576 females to age-, sex- and strain-matched transgenic negative littermates (WT).

All procedures were carried out in accordance with the current European and Spanish regulations (86/609/EEC; RD1201/2005). This study was approved by the Ethical Committee of the University of Navarra (no. 137/010).

### rAAV administration

Eighteen-month-old male C57BL/6 (12 per group), 12-month-old female Tg2576 (15 per group) or 4-month-old female Tg2576 (6 per group, for electrophysiology measurements) were anesthetized with ketamine/xylazine (80/10 mg/kg, i.p.) and placed in a stereotactic frame. The scalp was shaved and a longitudinal incision was made along the midline of the skull. The dorsal surface of the skull was then exposed, and two burr hole were drilled above the infusion sites. 1.0 μL of virus suspension or saline solution (sham mice) was infused to the CA1 region of the hippocampus (−2.0 mm AP, ±1.7 mm ML, −2.0 mm DV to bregma) according to the Paxinos and Watson mouse brain atlas (1998). A 1-μl Hamilton syringe was used for the infusion (Hamilton Co., Reno, NV). The infusion rate was 0.2 μl/min, and the needle remained in place for 5 min after the infusion for vector absorption. The same procedure was used for the infusion in the other hemisphere. Finally, the site was stitched closed.

### Human AD samples and controls

A total of 24 brain samples from the Navarra Health Service /Osasunbidea's Research Biobank were included in the study. The samples were obtained from 15 patients with clinical diagnosis of dementia and 9 elderly normal controls. Patients with AD met criteria proposed by the National Institute on Aging and the Alzheimer's Association workgroups for AD dementia with documented decline (McKhann*et al*, 2011). For all subjects, informed consent had been obtained from relatives before the removal of brain tissue at death and subsequent use of the material for research. This study was carried out in accordance with the Medical Ethics Committee of the University of Navarra. At autopsy, brains were removed and stored at −80°C until processing.

### Primary neuronal cultures

Primary neuronal cultures were derived from the hippocampus of embryonic day 16 (E16) Tg2576 or wild-type (WT) mice. Hippocampi were triturated using glass pipettes until neurons were dissociated. Neurons were plated in serum-free neurobasal media with B27 supplement (Invitrogen, Gaithersburg, MD) and 2 mM l-glutamine on poly-L-lysine-treated (0.1 mg/ml; Sigma) 12-well plates. In Tg2576 neuronal cultures, to maintain increased levels of extracellular Aβ, media were not changed. Primary neurons were viable for > 3–4 weeks under our culturing conditions. Genotyping was performed on cerebellum from the same embryo.

Tg2576 and non-transgenic hippocampal cultures at 21 DIV were collected in a cold lysis buffer with protease inhibitors (0.2 M NaCl, 0.1 M HEPES, 10% glycerol, 0.2 M NaF, 2 mM Na_4_P_2_O_7_, 5 mM EDTA, 1 mM EGTA, 2 mM DTT, 0.5 mM PMSF, 1 mM Na_3_VO_4_), left on ice for 30 min and centrifuged at 14,000 *g* for 20 min at 4°C. The supernatant was aliquoted and stored at −80°C. Total protein concentrations were determined using the BioRad Bradford protein assay (BioRad Laboratories, CA).

For viral transduction of Tg2576 neuronal cultures, neurons were cultured for 10 days in 12-well plates prior to transduction with 1.13 × 10^8^ viral transducing units per well. Blocking anti-IGF2R antibody (1:100, Santa Cruz Biotechnology) or the selective IGF1R antagonist JB1 (500 pg/μl, Cymit Química, Barcelona) was used 24 h before collecting the media to block the IGF2R or IGF1R. The medium was collected 72 h after the virus infection and stored at −80°C until Aβ42 was determined by ELISA.

For Aβ_42_ measurements in WT neurons, hippocampal cultures at 20 DIV were treated with 50% Tg2576 conditioned medium (CM) as a natural source of Aβ_42_, protease inhibitor (PI) cocktail (Complete Protease Inhibitor Cocktail, Roche Diagnostics, Mannheim, Germany) and/or rabbit polyclonal antibody anti-IGF2R (1:100, Santa Cruz Biotechnology). The medium was collected after 48 h and stored at −80°C until Aβ_42_ was determined by ELISA.

### Cell line cultures

BNL CL.2 and HepA129 cell lines were obtained as a gift from the Dr. Fortes at CIMA (Dpto of Gene Therapy and Hepatology). Cells were cultured in 12-well plates to 90% confluence at 37°C in an atmosphere of 5% CO_2_. BNL CL.2 cells were grown in Dulbecco's modified Eagle's medium (DMEM) supplemented with Glutamax (Gibco, Invitrogen, CA), 100 units/ml penicillin/streptomycin and 10% foetal bovine serum (FBS). HepA129 cells were grown in Roswell Park Memorial Institute (RMPI) 1640 medium supplemented with Glutamax (Gibco, Invitrogen, CA), 100 units/ml penicillin/streptomycin and 10% FBS.

Cells were exposed to Tg2576 conditioned medium for 1, 4 and 24 h, and the medium was collected and stored at −80°C until Aβ_42_ measurements by ELISA.

### Contextual fear conditioning

The behavioural procedure involved three phases: habituation, training and testing. During habituation phase, mice were habituated to the conditioning box (context) for 5 min with no stimuli presented. Twenty-four hours later (training phase), mice were placed in the training chamber and allowed to explore for 2 min, and afterwards, a footshock (0.3 mA) unconditioned stimulus was administered (2 s) and 30 s after mice were returned to their home cage. A stronger paradigm of training (2 CS-US pairings, separated by a 10-s resting interval) was used for the group of transgenic mice.

Mice were returned to the conditioning chamber 24 h after training and allowed to explore the context for 3 min, during which freezing behaviour was recorded (contextual long-term memory). Freezing scores were expressed as percentages. The conditioning procedure was carried out in a StartFear system (Panlab S.L., Barcelona, Spain) that allows recording and analysis of the signal generated by the animal movement through a high-sensitivity Weight Transducer system. The analogical signal is transmitted to the FREEZING and STARTLE software modulated through the load cell unit for recording purposes and posterior analysis in terms of activity/immobility.

### Morris water maze

To evaluate the working and spatial memory function in response to AAV-IGF1/IGF2 injection, the Morris water maze test was performed, as previously described (Ricobaraza*et al*, 2009). Mice underwent visible-platform training for three consecutive days (eight trials per day) using a platform raised above the surface of the water. No visible cues were present during this phase. The visible-platform phase was followed by hidden-platform training (visible cues present) during which mice were trained to locate a platform submerged 1 cm beneath the water surface for 8 consecutive days (four trials per day). In both visible- and hidden-platform versions, mice were placed pseudo-randomly in selected locations. Each trial was terminated when the mouse reached the platform or after 60 s, whichever came first. After each hidden-platform trial, mice remained on the platform for 20 s. Twenty hours after the 12th, 24th and 32nd trials, all mice were subjected to a probe trial in which they swam for 60 s in the pool with no platform. All trials were monitored by a camera above the centre of the pool connected to a SMART-LD program (Panlab S.L., Barcelona, Spain) for subsequent analysis of escape latencies (time required to reach the platform), swimming speed, path length and percentage time spent in each quadrant of the pool during probe trials. All experimental procedures were performed blind to groups.

### RNA extraction and PCR

Total RNA from hippocampus and prefrontal cortex of mice was isolated using Trizol reagent (Sigma-Aldrich, St Louis, MO). The RNA was treated with DNase at 37°C for 30 min and reverse-transcribed into cDNA. To detect the expression of viral IGFs, conventional PCR was performed with oligonucleotide primers specific to viral IGFs; thus, the 5′ primer was designed against the 5′ end of the corresponding IGF cDNA and a 3′ primer aligned to the 3′ UTR, poly-adenylation signal sequence of the recombinant IGF, which is common for both viral IGF2 (forward: 5′-GCTTGCCAAAGAGCTCAAAG-3′; reverse: 5′-ACTCGAGGTCGAGGCCAGA-3′), and viral IGF1 (forward: 5-CCCCACTGAAGCCTACAAAA-3′; reverse: 5′-ACTCGAGGTCGAGGCCAGA-3′). The PCR product was analysed on a 1% agarose gel containing SYBR Safe DNA gel stain (Invitrogen, Carlsbad, CA) under ultraviolet light.

For the quantification of IGFs expression, quantitative real-time PCR assays were performed in triplicate using Power SYBR Green PCR Master Mix (Applied Biosystems, Warrington, UK), and specific primers to mouse IGF2 (forward: 5′-CGCTTCAGTTTGTCTGTTCG-3′; reverse: 5′-GGAAGTACGGCCTGAGAGGTA-3′), mouse IGF1 (forward: 5′-GGACCGAGGGGCTTTTACTTC-3′; reverse: 5′-AGTCTTGGGCATGTCAGTGTGG-3′) and β-actin (forward: 5′-CCTGACAGACTACCTCATGAAG-3′; reverse: 5′-CCATCTCTTGCTCGAAGTCTAG-3′) was used as internal control. Real-time PCR was done using an ABI Prism 7300 sequence detector (Applied Biosystems, Foster City, CA), and data were analysed with Sequence Detection software v. 3.0. (Applied Biosystems).

### Immunofluorescence

Under xylazine/ketamine anaesthesia, 20-month-old Tg2576 mice from each group (*n* = 4) were transcardially perfused with saline and 4% paraformaldehyde in phosphate buffer (PB). After perfusion, the brain was removed, post-fixed for 1 h at 4°C in the same fixative solution and cryoprotected overnight at 4°C in 30% sucrose in PB. Coronal microtome sections (30 μm thick) containing the hippocampal formation were collected and processed for immunostaining. Brain sections were incubated in 70% formic acid for 10 min to expose the epitope prior to incubation with blocking solution (PBS containing 0.5% Triton X-100, 0.1% BSA and 2% normal goat serum) for 2 h at room temperature. Sections were then incubated for 24 h at 4°C with mouse monoclonal 6E10 (against amino acids 1–17 of Aβ peptide, 1:200, Covance) diluted in blocking solution. After washing with PBS, the brain sections were incubated with the secondary antibody (1:200, Alexa Fluor 488 goat anti-mouse highly cross-absorbed Molecular Probes, Eugene, Oregon) for 2 h at room temperature in the dark. Fluorescence signals were analysed on a confocal microscope (LSM 510 Meta, Carl Zeiss, Germany) using a Plan-neofluar 40×/1.3 oil DIC objective. Sections were evaluated in Z-series (0.4 mm steps) using LSM 510 Meta software.

Amyloid deposition was quantified using Aβ immunostaining (monoclonal anti- Aβ 6E10) and the AnalySIS Image System (Ribe*et al*, 2005). Video images were captured of each region of interest on 30-μm sections, and a threshold of optical density was obtained that discriminated staining from background. Manual editing eliminated artefacts. The amyloid burden, defined as the total percentage of area covered by amyloid deposits over three sections, was calculated for hippocampus.

### Protein extracts

Brain samples were homogenized in a cold lysis buffer with protease inhibitors (0.2 M NaCl, 0.1 M HEPES, 10% glycerol, 0.2 M NaF, 2 mM Na_4_P_2_O_7_, 5 mM EDTA, 1 mM EGTA, 2 mM DTT, 0.5 mM PMSF, 1 mM Na_3_VO_4_), left on ice for 30 min and centrifuged at 14,000 *g* for 20 min at 4°C. To analyse IGF2 expression in mice, hippocampi were homogenized in a buffer containing 0.32 M sucrose, 5 mM HEPES pH 7.4, 1 mM MgCl2, 1 mM EDTA, 1 mM NaHCO3, 0.1 mM PMSF and protease inhibitors. The supernatant was aliquoted and stored at −80°C. Total protein concentrations were determined using the BioRad Bradford protein assay (BioRad Laboratories, CA).

### Western blot analysis

Protein samples were mixed with 6× Laemmli sample buffer, resolved onto SDS-polyacrylamide gels and transferred to nitrocellulose membranes. The membranes were blocked with 5% milk in TBS followed by overnight incubation with the following primary antibodies diluted in 2.5% milk in TBS: rabbit polyclonal anti-IGF2 (1:500, Abcam) and anti-β-actin (1:100,000 Sigma-Aldrich, St. Louis, USA). Following two washes in TBS/Tween-20 and one in TBS alone, the immunolabelled protein bands were detected with an HRP-conjugated anti-rabbit (1:5,000, Santa Cruz) or anti-mouse (1:5,000, Santa Cruz) antibody. Finally, antibody binding was visualized by enhanced chemiluminescence system (ECL; GE Healthcare Bioscience) and autoradiographic exposure to Hyperfilm™ECL (GE Healthcare Bioscience). Quantity One™ software v.4.6.3 (Bio-Rad) was used for protein quantification.

### Dendritic spine measurements

A modified Golgi-Cox method was used (Glaser and Van der Loos, 1981). Briefly, brains were removed from the skull and placed in Golgi-Cox solution (1% potassium dichromate, 1% mercury chloride, 0.8% potassium chromate) for 3 weeks. Brains were then washed in water and 90% ethanol, and 200-μm-thick slices were cut on a vibratome and soaked in 16% ammonia solution for 1 h. After fixation in 1% sodium thiosulphate solution, slices were dehydrated and mounted with DPX. Slides were coded before quantitative analysis, and the code was broken only after the analysis was completed. To be selected for morphometric analysis, Golgi-impregnated pyramidal cells had to be located within the CA1 and have consistent impregnation throughout untruncated dendrites. CA1 dendrites were classified into primary and secondary dendrites: dendrites directly originating from cell soma were classified as primary dendrites, and those originating from primary dendrites were classified as secondary. Each selected neuron was captured in a Nikon Eclipse E600 light microscope, and images were recorded at a resolution of 1,000–1,500 dots per inch (dpi) using a digital camera (Nikon DXM 1200F). Data for the statistical analyses were obtained from 100× pictures of a random sample of apical secondary dendrites taken between 100 and 200 μm apart from the soma, where spine density is relatively uniform in CA1 pyramidal neurons (Megias*et al*, 2001). For each mouse (*n* = 3–4 per group), 9 neurons (three dendritic segments were measured per neuron, at least 30 μm long) were analysed.

### Slice preparation and electrophysiology

Seven to 8-month-old mice (3–4 months after the AAV injection) were anesthetized with ketamine (80 mg/kg, i.p.) and xylazine (10 mg/kg i.p.) and intracardially perfused in an ice-cold oxygenated cutting solution (200 mM of sucrose, 20 mM glucose, 0.4 mM CaCl_2_, 8 mM MgCl_2_, 2 mM KCl, 1.3 mM NaH_2_PO_4_, 26 mM NaHCO_3_, 3 mM pyruvate and 2 mM of kynurenic acid (pH 7.3). Brain was quickly dissected, and slices of 350 μm were made with a Leica vibratome and then kept in oxygenated artificial CSF [125 mM NaCl, 2.5 mM KCl, 2.5 mM CaCl_2_, 1.3 mM MgSO_4_, 1.25 mM NaH_2_PO_4_, 26 mM NaHCO_3_ and 20 mM glucose (pH 7.4)] at 33°C for 30 min in the presence of kynurenic acid (2 mM) and left to rest for 30 min at room temperature without kynurenic acid. Slices were used within a maximum of 6 h after cutting.

Brain slices were transferred to a recording chamber, in which they were continuously superfused with oxygenated (95% O_2_ and 5% CO_2_) artificial CSF. The patch pipettes were filled with a solution caesium methanesulfonate-based solution (140 mM CsMeSO_3_, 2 mM MgCl_2_, 4 mM NaCl, 0.2 mM EGTA, 5 mM P-creatine, 3 mM ATPNa_2_, 0.3 mM GTP, 10 mM HEPES). Bicuculline (10 μM) and TTX (1 μM) were present in the superfusate of all experiments. Cells were clamped at −70 mV, and mEPSCs were recorded for 15 min in both CA1 and CA3 pyramidal cells. The access resistance of the cells was < 20 MΩ, and recordings were discarded from analysis if the resistance changed by > 20% over the course of the experiment. Recordings were made using an EPC 10.0 amplifier (HEKA Elektronik) and were filtered at 0.5–1 kHz, digitized at 1–5 kHz.

The amplitude and the time interval between events were measured, and the values of the first 100 events and intervals of every cell were plotted using a threshold of 6 pA. For each condition, 10 cells were recorded, from 3 to 5 animals.

### Determination of Aβ levels

Cortical Aβ_42_ and Aβ_40_ levels were measured by using a sensitive sandwich ELISA kit (Invitrogen, Camarillo, CA). Cortical tissue was weighed and homogenized in 8× mass of cold-ice guanidine buffer (5M guanidine HCl/50 mM Tris HCl pH 8.0). The homogenates were mixed for 4 h at room temperature and were diluted 1:50 in Dulbecco's phosphate-buffered saline containing 5% BSA and 0.03% Tween-20 (DPBS-BSAT) supplemented with protease inhibitor cocktail (Complete Protease Inhibitor Cocktail, Roche Diagnostics, Mannheim, Germany) followed by centrifugation at 16,000 *g* for 20 min at 4°C. The supernatant was diluted and loaded onto ELISA plates in duplicate. For Aβ_42_ measurement from conditioned medium, it was directly diluted and loaded onto ELISA plates in duplicate. The assays were performed according to the manufacturer's instructions, and Aβ standards were prepared in a buffer with the same composition as the samples.

### Data and statistical analyses

The results were processed for statistical analysis using SPSS package for Windows, version 15.0 (SPSS, Chicago, IL, USA). Unless otherwise indicated, results are presented as mean ± standard error of the mean (SEM). Normal distribution of data was checked by the Shapiro–Wilk test. Unpaired two-tailed Student's*t*-test was used in case two groups were compared. Normally distributed variables for more than two groups were analysed using one-way ANOVA followed by Scheffe's*post hoc* test. Non-normally distributed were analysed using Kruskal–Wallis test. In the invisible-platform phase of Morris water maze, Friedman's test was performed to determine the intra-group comparisons over trials. Electrophysiological data (cumulative distribution of the peak amplitude and intervals of mEPSCs) were analysed using Kolmogorov–Smirnov (KS) test.

**The paper explained**

**Problem**

Alzheimer's disease (AD) is the most common cause of dementia in the elderly and with no proven effective treatment currently. Agents that reverse synapse impairments and Aβ accumulation represent promising therapeutic candidates for the treatment. An important role for hippocampal IGF2 in brain plasticity and learning and memory has been reported, and therefore, it may represent a new therapeutic candidate for AD treatment.

**Results**

We report that the endogenous levels of IGF2 decrease in the hippocampus with aging and in pathological conditions related to AD. Thus, we employed an adeno-associated viral (AAV) vector system to deliver IGF2 in the hippocampus of the transgenic mouse model Tg2576 to test its effect on the behavioural, cellular and synaptic phenotypes typical of AD. We demonstrated that the overexpression of hippocampal IGF2 restores memory and synaptic transmission deficits related to AD. We also demonstrated that IGF2 facilitates Aβ degradation in Tg2576 mice brain, which may be mediated by IGF2R.

**Impact**

Our results strongly suggest that increasing IGF2 in brain has a therapeutic effect in AD pathogenesis. This is in accordance with other studies that identify IGF2 as a natural memory boost, an alternative to purely pharmacological agents. However, in one side, IGF2 is related to cancer because it favours cell growth. Since IGF2 and IGF2R are known to be imprinted, it would be interesting to know whether the epigenetic changes triggered during learning can also induce IGF2 expression. Besides the gene therapy used in this study, it would be helpful to discover the mechanism by which neurons can increase IGF2 production in the brain in AD conditions.
