# Supplementary material for: Insulin-like growth factor 2 reverses memory and synaptic deficits in APP transgenic mice
Source: EMBO Mol Med. 2014 Aug 7;6(10):1246–62. doi: 10.15252/emmm.201404228 (PMC4287930; doi:10.15252/emmm.201404228)
Supplement: Supplementary file 1 [file emmm0006-1246-sd1.pdf]

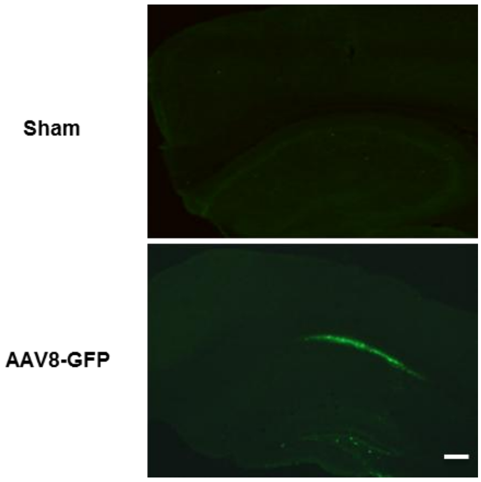

Figure S1. Two months after the intrahippocampal administration of AAV8-GFP, GFP was detected in the pyramidal cells of CA. No signal was detected in sham-injected animals. Scale bar = 20 μm.

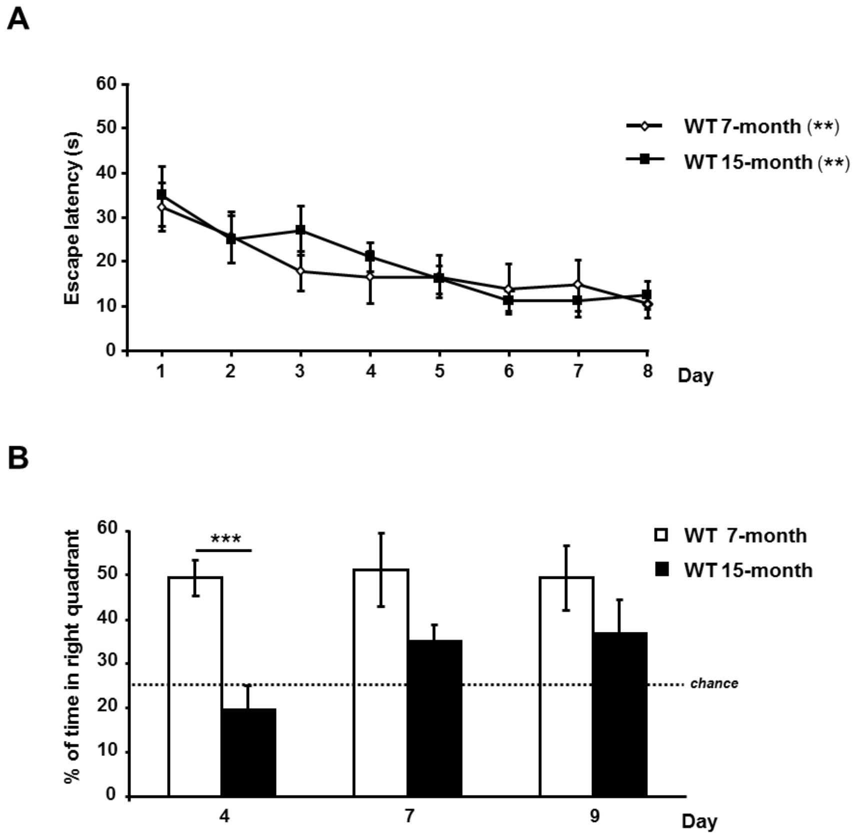

Figure S2. Aged wild type mice display impaired spatial memory in the Morris water maze.

A. Escape latency to the hidden platform in the Morris water maze test for 7 month-old and 15 month-old WT mice. Latency to reach the platform decreased in both groups as the training sessions progressed (non-parametric Friedman test, n = 7-9, ** p = 0.002 WT 7-month, ** p = 0.008 WT 15-month).

B. Percentage of time spent searching for the target quadrant in the probe test (days 4, 7 and 9). On day 4, 15-month-old WT mice performed significantly more poorly than 7 month-old WT mice (unpaired two-tailed Student's *t* test, n = 7-9, *** p = 0.0005).

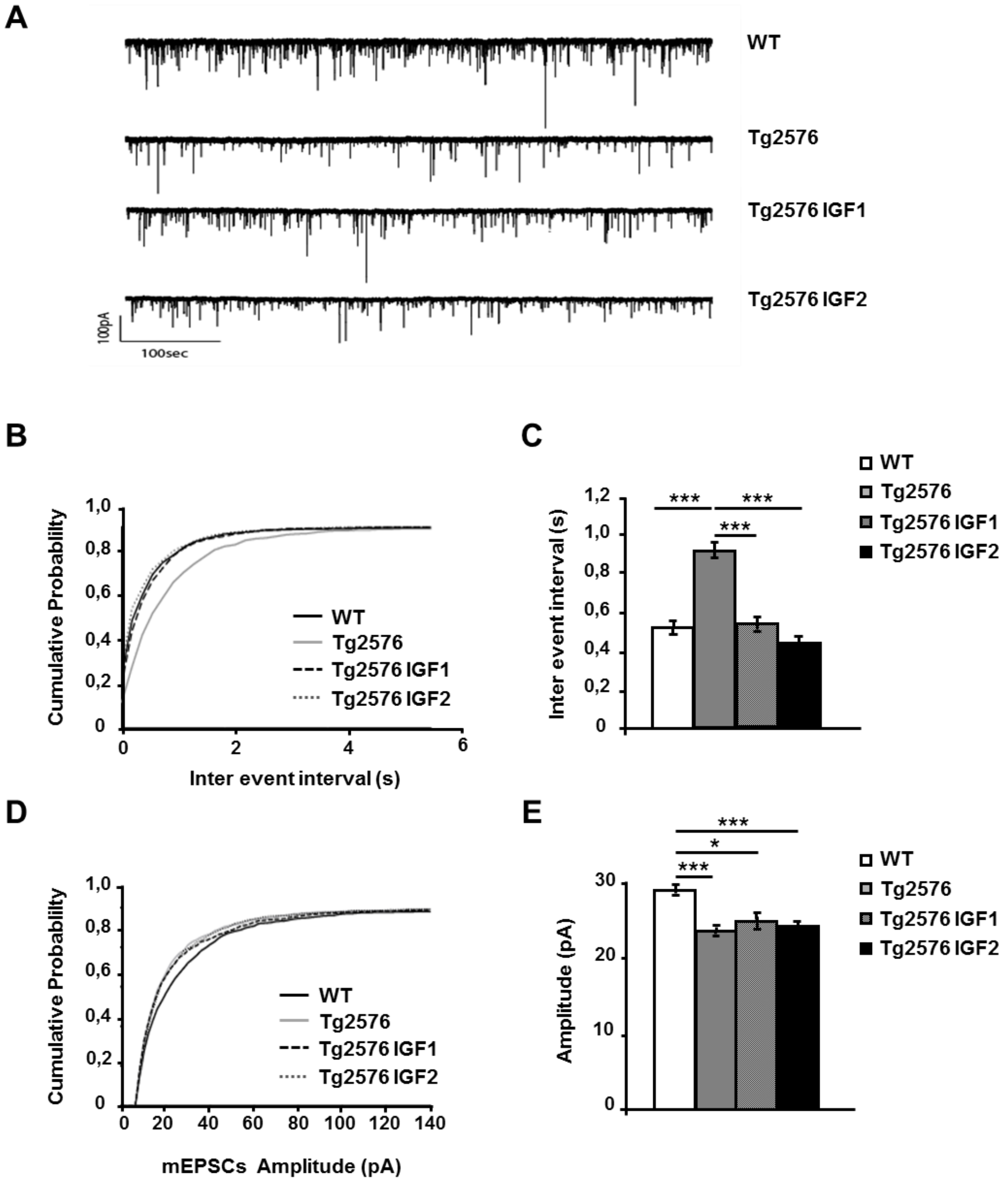

Figure S3. IGF1 and IGF2 treatment restores CA3 synaptic transmission to control levels.

A. Miniature EPSCS were recorded from CA3 pyramidal cells in animals in 7-month-old animals four months after treatment (n = 10 in all conditions). Cells were held at -70mV in the presence of bicuculline (10µM) and TTX (1µM). Representative traces of

spontaneous AMPA EPSCs were isolated from the first 15 minutes after whole cell configuration.

B. Cumulative probability analysis of the distribution of inter-event intervals between mEPSCS with very significant difference between WT and Tg2576 (non parametric Kolmogorov-Smirnov test, n = 10, $p < 0.0001$). Treatment with IGF1 and IGF2 rescued the frequency of mEPSCS to values not different from control animals ($p < 0.0001$ Tg2576 vs Tg2576 IGF1, $p < 0.0001$ Tg2576 vs Tg2576 IGF2).

C. Average mEPSCs interval between the first 100 events of 10 cells per treatment (one-way ANOVA followed by Tukey's *post hoc* test, n = 10, *** $p < 0.001$).

D. Cumulative probability analysis of mEPSCS amplitude with difference between Tg2576 and WT (non parametric Kolmogorov-Smirnov test, n = 10, $p < 0.0001$). The mEPSCs amplitude of the treated groups is not rescued to values of the WT animals ($p = 0.4324$ Tg2576 vs Tg2576 IGF1, $p = 0.6476$ Tg2576 vs Tg2576 IGF2).

E. Average amplitude of the first 100 events of 10 cells per treatment (one-way ANOVA followed by Tukey's *post hoc* test, n = 10, * $p < 0.05$, *** $p < 0.001$).

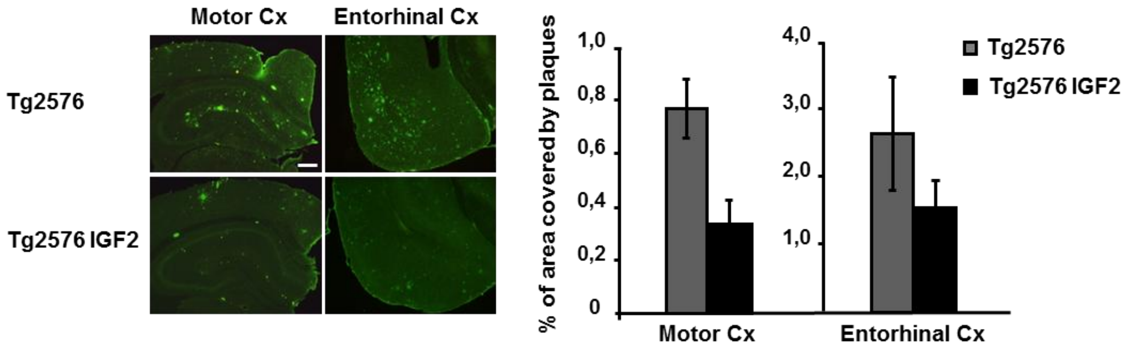

Figure S4. IGF2 decreases amyloid burden in cortical areas in 20-month-old Tg2576 mice.

Representative brains sections of sham- (Tg2576) and AAV-IGF2 (Tg2576 IGF2) -treated Tg2576 mice are shown (left panel). Scale bar = 100 µm. Amyloid burden quantification (right panel). Multiple extracellular deposits stained with 6E10 antiserum were detected in Tg2576 mice. Amyloid burden is reduced in the motor cortex and entorhinal cortex of Tg2576 IGF2 mice, but no significant differences were found between groups (n = 3-5).

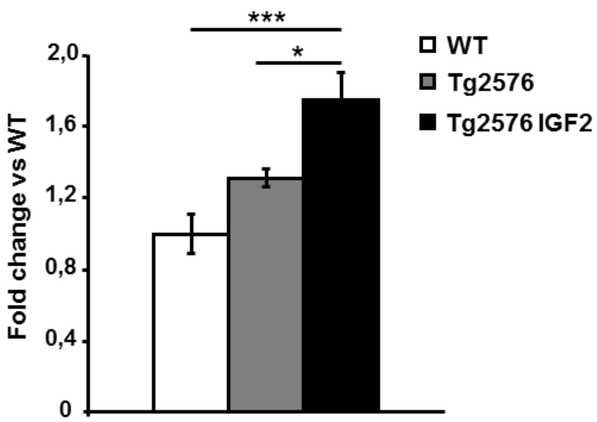

Figure S5. AAV-IGF2-injected mice showed an increase in IGF2R expression in the hippocampus.

Quantification of the relative expression of IGF2R transcripts was determined by real time PCR analysis using primers designed against the corresponding cDNA of IGF2R. IGF2-injected transgenic mice (Tg2576 IGF2) showed a robust increase in IGF2R compared to WT animals (WT) and to sham-injected transgenic mice (Tg2576 mice). Data are expressed as the fold change (mean ± SEM) with respect to the WT (one-way ANOVA followed by Scheffe's *post hoc* test, n = 5-6, *** p = 0.0003 WT vs Tg2576 IGF2, * p = 0.015 Tg2576 vs Tg2576 IGF2).
