# Supplementary material for: Insulin-like growth factor 2 reverses memory and synaptic deficits in APP transgenic mice
Source: EMBO Mol Med. 2014 Aug 7;6(10):1246–62. doi: 10.15252/emmm.201404228 (PMC4287930; doi:10.15252/emmm.201404228)
Supplement: Supplementary file 2 [file emmm0006-1246-sd2.pdf]

Insulin-like growth factor 2 reverses memory and synaptic deficits in APP transgenic mice.

Maria Pascual-Lucas, Silvia Viana da Silva, Marianna Di Scala, Carolina Garcia-Barroso, Gloria Gonzalez-Aseguinolaza, Christophe Mulle, Cristina M. Alberini, Mar Cuadrado-Tejedor and Ana Garcia-Osta

Corresponding author: Ana Garcia-Osta, CIMA, University of Navarra

Review timeline:

Submission date:	07 May 2014
Editorial Decision:	30 May 2014
Revision received:	16 June 2014
Editorial Decision:	30 June 2014
Revision received:	11 July 2014
Accepted	16 July 2014

Transaction Report:

(Note: With the exception of the correction of typographical or spelling errors that could be a source of ambiguity, letters and reports are not edited. The original formatting of letters and referee reports may not be reflected in this compilation.)

Editor: Céline Carret

1st Editorial Decision 30 May 2014

Thank you for the submission of your manuscript to EMBO Molecular Medicine. We have now heard back from the three referees whom we asked to evaluate your manuscript and discussed within the editorial team about the next step.

As you will see from the reports below, the referees find the topic of your study to be of interest. However, they all raise substantial concerns on your work, being of an experimental nature (referees 1 and 2) but also regarding the conceptual advance (referee 3). Your study was submitted after the release of the article mentioned by referee 3; while due to its interest and putative suitability for publication in EMBO Molecular Medicine, the manuscript was sent out for external review, in light of referees' comments, we would like to give you a chance to address all issues. However, as I am sure you realise, time is critical and publication should be fast. As such, we would like to give you 2 weeks and would then opt for a fast track acceptance. I hope you would agree.

I should emphasise that EMBO Molecular Medicine policy allows only a single round of revision and that, therefore, acceptance or rejection of the manuscript will depend on the completeness of your responses included in the next, final version of the manuscript. I realize that addressing the referees' comments in full in 2 weeks would involve a lot of additional experimental work and I am uncertain whether you will be able (or willing) to return a revised manuscript within this deadline and I would also understand your decision if you chose to rather seek rapid publication elsewhere at this stage.

I look forward to seeing a revised form of your manuscript within 2 weeks.

Should you find that the requested revisions are not feasible within the constraints outlined here and choose, therefore, to submit your paper elsewhere, we would welcome a message to this effect.

***** Reviewer's comments *****

Referee #1 (Comments on Novelty/Model System):

Technical quality and novelty are high. The medical impact was judged as only medium as there are no data on pharmacological activation of IGF2 in the manuscript. The model system, mice, is very adequate.

Referee #1 (Remarks):

In the article entitled "Insulin-like growth factor 2 reverses memory and synaptic deficits in APP transgenic mice", Pascual-Lucas et al. examine the potentially beneficial effects of exogenous IGF2 administration against age and Alzheimer's Disease (AD)-related pathologies. Namely, they show that virus-mediated overexpression of IGF2 is beneficial against age and AD-related cognitive decline in 20 month old mice and TG2576 mice, respectively; that such expression is further beneficial in increasing spine density and in decreasing amyloid- levels, the latter both in vivo and in vitro.

Overall, this is well-structured and well-conceived study, the results obtained are for the most part (see exceptions below) clear and the data is compelling. In line with previously published reports indicating a beneficial role for IGF2 of enhancing memory consolidation and extinction, as well as of promoting synapse formation and maturation, the present study adds to the growing evidence of the importance of IGF2 in the healthy and the diseased brain. We recommend this study for publication in EMBO Molecular Medicine, provided that the following major and minor points are properly addressed.

Experiments pertaining to figure 1:

- The reader would benefit from a lower magnification image depicting GFP expression throughout the entire hippocampal area in order to gauge its spreading following viral injections.
- In the results section describing the western blot images, it is not correct to state "quantitative", as no quantification was conducted on the western blots.

Experiments pertaining to figure 2:

- It would have been interesting to see (or at least be informed of) the memory performance of IGF2-injected aged animals vs young animals (e.g., 6 or 12 months of age). The authors may want to run an additional behavioral experiment on this age group and state the results as supplementary data. There is no need to repeat all the experimental groups.
- Similarly, what is the effect of age on endogenous IGF2 levels?
- The results of the experiments in figure 2 should be mentioned in the abstract.

Experiments pertaining to figure 4:

- Figure 4D is missing in the figure deck.

Experiments pertaining to figure 5:

- In the Morris Water Maze training phase, WT animals seem to start out with a better performance than all other groups and their amelioration over the training days is somewhat marginal. Is the difference between WT and the other groups on Day 1? And is the decrease over time in the WT really significant? If the answer to the first question is yes, this might constitute a confounding factor, which the authors need to properly address.
- In figure 5D, it is important to show the performance of all the groups in all quadrants of the maze as supplementary data.

Experiments pertaining to figure 6:

- It is not quite obvious why in this set of experiments the authors dealt with much younger animals than in the previous ones.

Experiments pertaining to figure 7:

- Figure 7A: Is this reduction significant?
- IHC and ELISA experiment need to be performed and shown in both the cortical areas and the hippocampus.

Experiments pertaining to figure 8:

- How efficient is the blocking of IGF1 with JB1?
- The IGF2-mediated effect of IGF2 is not convincing, but becomes more convincing with the supplementary data and the use of additional cell lines. Perhaps these figures should be combined.

Other comments:

- Figures 1 and 2 should be combined.

Referee #2 (Comments on Novelty/Model System):

The details for the evaluation are outlined in the "Remarks send to author" section.

Referee #2 (Remarks):

The manuscript by Pascual-Lucas and colleagues presents interesting data pertaining the role of IGF2 in learning and memory and in Alzheimer's disease (AD). The work essentially consists of the use of an AD mouse model to establish the efficacy of AAV-mediated IGF2 overexpression in learning-relevant regions of the brain to revert cognitive deficits and associated synaptic phenotypes. While the data presented in the manuscript address a highly interesting topic, there are some technical and conceptual concerns that need to be addressed.

Major points:

1) In Figure 1, authors show an image of the efficacy of AAV-GFP injections, but no image of the AAV-IGF2 injection. Even if the authors decided to use the IGF2 cDNA alone and not fused to GFP (which might have been the more correct comparison to the controls, see also next point), it would be desirable to have an IHC image to see the spread/coverage and quality of the injection with the AAV-IGF2 vector as well. The authors do provide a WB image (Fig 1C), but there are two issues related with it: on the one hand, there is no loading control making it difficult to compare the groups. More importantly, if the antibody used for detection was a-IGF2, I do not understand why there is no band in the sham groups, since IGF2 is produced in the hippocampus. Same is true for figure 4D. My feeling is that it would have been much better to overexpress IGF2 that could be distinguished somehow form endogenous IGF2. For example if the GFP tag is considered to be to big, a myc tag would have been suitable. Since all subsequent results are based on this data it is important to address this point somehow.
2) Although sham-injected animals are put through the same procedure as AAV-injected animals, the question remains whether sham injections are really comparable to an AAV-mediated overexpression. It might have been more convincing to either clone IGF2 fused to GFP or with an IRES element to grant coexpression of GFP. In the setup chosen by the authors, it might have been preferable to use a GFP-injected control to better control for AAV infection and protein overexpression effects. One possible control here is to prove that sham or GFP injections do not alter the basal levels of IGF2 and do not alter basal behavior.
3) On page 6 of the manuscript, it is stated that "IGF2 treatment reverses ageing-related defects...".

While this may certainly be the case, this claim cannot be made in light of the data presented, as no young control is provided for comparison. This is also claimed in the discussion on page 15
4) Relating to the point above, one could claim that a 30% freezing is relatively low, but actually, if one compares the freezing levels of old sham-injected mice with that of the wt controls presented in subsequent figures (which are also old), it is quite remarkable that aged wt mice have an 80% freezing. Indeed, an 80% freezing is quite unlikely after a 0.3mA shock. Authors should comment on both these issues.
5) On page 6 it is claimed that "although all the groups were able to remember the platform location...". This seems unlikely to be true for WT animals on the 4th day probe test. Please correct or provide statistical measure.
6) It would be helpful to get an explanation, why the MWM data from part 2 and part 5 show completely different baseline levels of escape latency, though the conditions for the wild-type control mice seem to be the same (assuming, that they are of the same age; unfortunately no information are given in part 2 about that)
7) The finding that injection of the IGF2 AAV in the hippocampus reduces amyloid load in the cortex is quite fascinating. However at present there is no good explanation for this phenotype. The authors mention the spread of the virus from the hippocampus to the prefrontal cortex but as pointed out above (point 1) the characterization of the AVV- IGF2 is difficult and I skepctical if any conclusion can be drawn fomr the AAV-GFP image show in Fig 1A.

Minor points:

1) On page 7, consider rephrasing the description of the culture data experiments. As it is now, it reads as if the Tg2567 cultures are to be treated with Ab as well.
2) Given that the line is available, authors should provide a measure of IGF2 protein levels in the brain of Tg2567 animals. Why go the trouble of looking in culture when it can be done in vivo and is more biologically relevant?
3) On page 11, authors claim that "only partially IGF1... reverses memory deficits". This is not really true for IGF1, as it is only seen in the fear conditioning and even there not fully. Please consider rephrasing to be more specific.
4) Sentences are sometimes really long and hard to read. There are some orthographical mistakes here and there (especially pertaining 3rd-person conjugation). Please revise.
5) Some figures are wrongly referred to (i.e. figure 1C which is referred to as 1D, figure 7 in the discussion part). Also, some data are described in the text without any reference to the explaining figure, most likely because the figure was once mentioned 3 paragraphs before.

Referee #3 (Comments on Novelty/Model System):

This manuscript is premature and fails to address principal issues (please see below). Novelty is also questionable as an article that reports similar findings has been recently published (Mellott et al., PLoS One. 2014 Apr 14;9(4):e94287). Accordingly, i do not think that this manuscript is suitable for publication in EMBO Molecular Medicine.

Referee #3 (Remarks):

How hormonal signaling cascades modulate proteotoxicity-associated maladies is a key question. In this study, Pascual-Lucas et al., tested whether over-expression of the IGF1 or IGF2 in the brain (by viral infection) alleviate aging and Alzheimer's disease (AD)-linked symptoms in mice. They found that IGF2 but not IGF1 protects from behavioral impairments and pathological hallmarks. They also validated some of their discoveries in human samples and cultured cells.
This is a potentially interesting study that reports important discoveries however key scientific and experimental aspects have not been adequately addressed.
First, the lively debate of whether IGF1 signaling protects from AD (Carro et al., Nat Med. 2002 Dec;8(12):1390-7) or promotes toxicity (Freude et al., FASEB J. 2009 Oct;23(10):3315-24 and Cohen et al., Cell. 2009 Dec 11;139(6):1157-69.). Secondly, the authors tested the expression of

IGF1 and IGF2 but did not provide evidences for possible functional outcomes. The claim that no IGF1 antibody is available (page 5, third paragraph) is not sufficient as they could test whether IGF-regulated transcription factors (e.g FoxO family members) change their cellular localization and whether known target genes are induced (for instance p27 or MnSOD).
The analysis of A levels is also fall short of being sufficient. It is widely accepted that small A oligomers, not fibrils are the most toxic species (Shankar et al., Nat Med. 2008 Aug;14(8):837-42 and Cohen et al., Cell. 2009 Dec 11;139(6):1157-69.). Thus, it is critical to employ size exclusion chromatography and ascertain the ration of monomers, oligomers and fibrils. Furthermore, the authors exclusively measured the amounts of A 42 while the ratio of A 40 to A 42 has been shown to be critically important.
I also find the claims that the over-expression of IGF2 improves synaptic activity not appropriately tested as no direct measurement of synaptic activity is presented.
The effect of IGF2 on cell viability should be tested in cultured cells by an adequate assay (for instance MTT assay).
In conclusion I think that this manuscript is premature.

1st Revision - authors' response 16 June 2014

Response to Reviewers

EMBO Molecular Medicine, Manuscript ID EMM-2014-04228-T: "Insulin-like growth factor 2 reverses memory and synaptic deficits in APP transgenic mice"

We thank the reviewers for their careful reading and helpful comments. Two of the reviewers found our manuscript to be interesting and considered it suitable for publication in EMBO Molecular Medicine. Reviewer#3 found the manuscript premature and lack of novelty. The three of them had a number of suggestions, which we address below. We have performed additional experiments as suggested, and now include extensive new data and clarification in the revised manuscript. We have revised the manuscript considerably to incorporate the new data, and also in response to several additional specific comments and insightful suggestions by the reviewers.

All these changes are detailed in the point-by-point responses below:

Reviewer #1 (Comments to the Author):

Experiments pertaining to figure 1:

- The reader would benefit from a lower magnification image depicting GFP expression throughout the entire hippocampal area in order to gauge its spreading following viral injections.

The reason for not including a lower magnification image depicting GFP is because it is difficult to distinguish the transduced neurons as clear as in the higher magnification picture. In the lower magnification picture (see Figure below) it is difficult to show neuronal expression of GFP in the cell bodies of pyramidal neurons and in the neuropil. Here we show the figure but it is not included in the manuscript. We think that the lower magnification picture does not add anything more than it does the pictures shown in the manuscript.

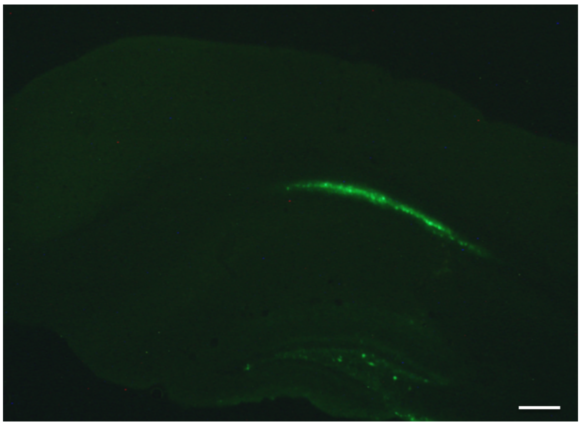

Scale bar: 20µm

- In the results section describing the western blot images, it is not correct to state "quantitative", as no quantification was conducted on the western blots.

We thank the referee for the comment; as recommended we have eliminated the word "quantitative" in the text when it was related to western blot analysis in Figure 1C.

Experiments pertaining to figure 2:

- It would have been interesting to see (or at least be informed of) the memory performance of IGF2-injected aged animals vs young animals (e.g., 6 or 12 months of age). The authors may want to run an additional behavioral experiment on this age group and state the results as supplementary data. There is no need to repeat all the experimental groups.

Thank you very much for this interesting comment. As suggested, we have performed a new Morris water maze experiment comparing 7-month to 15-month-old wild type mice and the data are included in supplementary Figure S1. Comparing Figure 1G and supplementary Figure S1B, you could observe that AAV-IGF2-injected mice behave similar to 7-month old wild type animals on probes on days 4 and 7, (reaching about 50% of time in the right quadrant) and, even better on probe on day 9 (63.4 % for IGF2 vs 49.29 % for 7-month-old). See page 6 in Results section.

- Similarly, what is the effect of age on endogenous IGF2 levels?

Thank you very much for this comment. In fact, due to your suggestion we tried to work out the technical conditions to detect endogenous IGF2 in the mice hippocampus. We have used a different lysis buffer (0.32 M sucrose, 5 mM HEPES pH 7.4, 1 mM $MgCl2$, 1 mM EDTA, 1 mM $NaHCO3$, 0.1 mM PMSF, protease inhibitors) and we have increased the amount of protein loaded in the western blot up to 100 µg. Finally, thanks to your comment, we have found very interesting results regarding the expression of IGF2 with the age and with the disease. We have found that the levels of IGF2 decreased with age. This observation further supports the potential involvement of IGF2 in the pathological detriment in age-dependent memory deterioration. Interestingly, we have also found that the levels of IGF2 decreased in Tg2576 mice hippocampus at the age of 7 month-old. The new data are now included in the manuscript.

- The results of the experiments in figure 2 should be mentioned in the abstract.

We thank the referee for the suggestion. We have now mentioned these results in the abstract.

Experiments pertaining to figure 4:

- Figure 4D is missing in the figure deck.

The referee is right. Sorry about this mistake. We have corrected the text.

Experiments pertaining to figure 5:

- In the Morris Water Maze training phase, WT animals seem to start out with a better performance than all other groups and their amelioration over the training days is somewhat marginal. Is the difference between WT and the other groups on Day 1? And is the decrease over time in the WT really significant? If the answer to the first question is yes, this might constitute a confounding factor, which the authors need to properly address.

We agree with the referee that the WT (Non-Tg) animals seem to start with a better performance. However, no significant differences were obtained on day 1 when compared the escape latency of WT group to the other groups. This subtle difference was also observed during the visible platform phase, where the escape latencies for these animals were also lower although no significant differences among groups were observed (see Figure below).

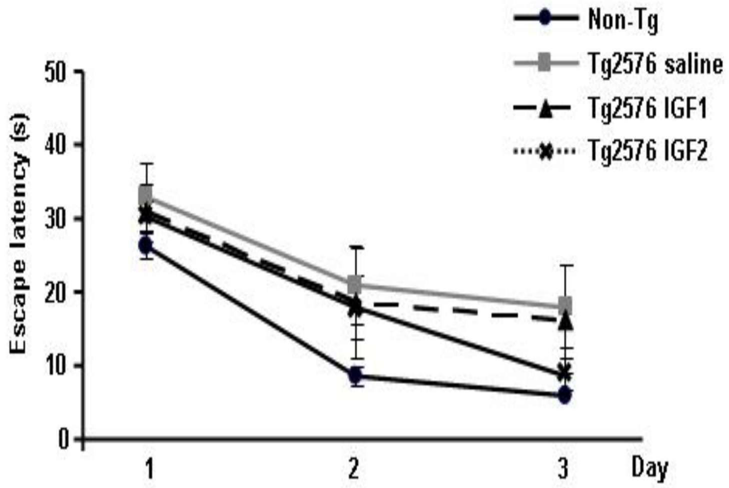

In any case, and, in relation to the second question, in the hidden platform phase (Figure 4C in the manuscript), the mean latency to reach the platform significantly decreased as the training sessions progressed in WT mice and in the Tg2576 IGF2 group ($P < 0.01$).

- In figure 5D, it is important to show the performance of all the groups in all quadrants of the maze as supplementary data.

Unfortunately we can not include these data in the manuscript. Usually we only measure the time spent in the target quadrant, so we did not keep the time spent in the other three quadrants. Sorry about this response.

Experiments pertaining to figure 6:

- It is not quite obvious why in this set of experiments the authors dealt with much younger animals than in the previous ones.

The main objective of these experiments was to understand if the reversal of spine loss by overexpression of IGF2 was promoting growth of active spines or rather inactive ones. For whole-cell patch clamp experiments it is crucial that the cells and brain slices are at best health, which can be better achieved when using 7-month-old animals. It has been described in literature that at this age there is already reduction of spine number in the Tg2576 mice hippocampus [1] [2], and specifically in CA1 pyramidal cells [3]. Our recordings of miniEPSCs confirm a reduction of events that can correspond to this phenotype.

Although the age of the animals is different, the period of overexpression was similar to the other experiments (3-4 month). With the electrophysiology data we show that 3 months of overexpression of IGF2 can rescue the number of events detected in CA1 cells to levels similar to the ones found in WT animals, confirming IGF2 treatment as a potential way to reverse synaptic loss in Alzheimer´s Disease.

Experiments pertaining to figure 7:

- Figure 7A: Is this reduction significant?

No, there is not a significant reduction in this case. The animals for immunofluorescence were only 3-5 for each group, thus, although a mark reduction on amyloid burden is appreciated in the graph, no significant differences were observed when statistical analysis was performed. Unfortunately, we do not have more tissue to increase the number of animals in this analysis.

- IHC and ELISA experiment need to be performed and shown in both the cortical areas and the hippocampus.

We did not perform the $A\beta_{42}$ ELISA quantification in hippocampus since specific extracts of guanidinium 5M are necessary to dissolve the $A\beta_{42}$ contained in the amyoid plaques and the hippocampus of the animals were employed to analyze IGF2 expression (RNA and a different lysis buffer to obtain protein extracts, see methods) and to quantify dendritic spine density. We have however quantified the plaque number by immunofluorescence in cortical areas as suggested. A marked reduction was observed in entorhinal and also in motor cortex in AAV-IGF2 mice compared to sham-injected animals. This new figure has been included as supplementary Figure 3S. Additional analysis of $A\beta_{40}$ levels has been also carried out using the guanidinium 5M extracts from cortical areas (see Figure 6B in the manuscript).

Experiments pertaining to figure 8:

- How efficient is the blocking of IGF1 with JB1?

JB1 is an analog of IGF1, which competes with IGF1 by binding the IGF1 receptor. It acts as a selective and potent IGF1R antagonist preventing its autophosphorylation, with no activity in IGF2R [4]. This analog is widely quick and highly specific method to investigate the role of IGF1R in

different cell types. It is also used to distinguish, and separate events that are specifically due to the activation of the IGF1 receptor from those due to other growth factor receptors.

- The IGF2-mediated effect of IGF2 is not convincing, but becomes more convincing with the supplementary data and the use of additional cell lines. Perhaps these figures should be combined.

Thank you for the suggestion. We have now combined the figures and the supplementary figure 3 became the Figure 7D.

Other comments:

- Figures 1 and 2 should be combined.

Thank you for the suggestion. We have also combined the figure 1 and 2.

Reviewer #2 (Comments to the Author):

Major points:

1) In Figure 1, authors show an image of the efficacy of AAV-GFP injections, but no image of the AAV-IGF2 injection. Even if the authors decided to use the IGF2 cDNA alone and not fused to GFP (which might have been the more correct comparison to the controls, see also next point), it would be desirable to have an IHC image to see the spread/coverage and quality of the injection with the AAV-IGF2 vector as well.

The reviewer is right. However, we choose the AAV for the advantages related to low immunogenicity and long-term expression. However they present also some disadvantages such as its cloning capacity, which is relatively limited and, therefore, we could not fit the IGF2/IGF1 plus GFP cDNA´s into this virus. Unfortunately, due to the nature as a paracrine molecule of IGF2 in the brain, and the limited sensibility we have not been able to obtain a successful image representing IGF2 expression in the brain, not even in the transduced animals.

- The authors do provide a WB image (Fig 1C), but there are two issues related with it: on the one hand, there is no loading control making it difficult to compare the groups. More importantly, if the antibody used for detection was a-IGF2, I do not understand why there is no band in the sham groups, since IGF2 is produced in the hippocampus. Same is true for figure 4D.

The reviewer is right. First of all, the loading control corresponding to western blot in Figures 1 and 3 were missing, sorry for that. Now, we provide new western blot analysis (see explanation below) and include the loading control.

It is highly difficult to detect endogenous IGF2 (protein) in the adult brain, whereas it is, easily detectable in neuronal culture (is produced in neurons). In our hands, by using western blot analysis, IGF2 was no detectable unless it was overexpressed (AAV-IGF2). We also tried to detect IGF2 in the hippocampus using ELISA assay (R&D Systems), and again we failed in the detection of the endogenous IGF2 protein.

However, after the referee's comments, we have tried to work out the technical conditions to detect endogenous IGF2 in the mice hippocampus. We have used a different lysis buffer (0.32 M sucrose, 5 mM HEPES pH 7.4, 1 mM MgCl2, 1 mM EDTA, 1 mM NaHCO3, 0.1 mM PMSF, protease inhibitors) and we have increased the amount of protein loaded in the western blot until 100 µg. Finally, thanks to the suggestions we have found very interesting results regarding the effect of age or disease on the expression of IGF2. We have found that IGF2 levels are reduced with age. This observation further supports the potential involvement of IGF2 in age-dependent memory deterioration. Interestingly, we have also found that IGF2 levels are reduced in the hippocampus of 7 month-old Tg2576 mice. At this age, these animals present an increase in amyloid levels and a decrease in dendritic spine density. Unfortunately we don't have available tissue to perform western blot analysis in older Tg2576 mice. These new data are now included in the manuscript (Figure 1D and Figure 2B). Importantly, these results support the idea that IGF2 administration may be beneficial for treating Alzheimer's disease.

- My feeling is that it would have been much better to overexpress IGF2 that could be distinguished somehow form endogenous IGF2. For example if the GFP tag is considered to be too big, a myc tag would have been suitable. Since all subsequent results are based on this data it is important to address this point somehow.

The referee is right. In our system, the only way to distinguished endogenous IGF2 from the overexpressed IGF2 is by RT-PCR using primers specific to viral IGFs. Thus, we designed a 5´ primer against the 5´ end of the corresponding IGF cDNA and a 3´ primer aligned to the 3' UTR, poli-adenilation signal sequence of the recombinant IGF. Using these primers we could confirm the viral source of the therapeutic genes (Figure 1B and Figure 3A).

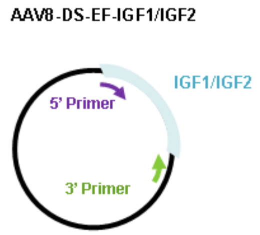

2) Although sham-injected animals are put through the same procedure as AAV-injected animals, the question remains whether sham injections are really comparable to an AAV-mediated overexpression. It might have been more convincing to either clone IGF2 fused to GFP or with an IRES element to grant coexpression of GFP. In the setup chosen by the authors, it might have been preferable to use a GFP-injected control to better control for AAV infection and protein overexpression effects. One possible control here is to prove that sham or GFP injections do not alter the basal levels of IGF2 and do not alter basal behavior.

As mentioned before, we choose the option of using AAV because of its safety, low immunogenicity and long-term expression in neurons, which make them suitable for the AD model. However, the biggest disadvantage is its relatively limited cloning capacity.

The referee is right, a good control for IGF would be AAV-GFP. However, according to the literature and reports showing no effect of IGF1 in memory, we chose AAV-IGF1 as a control for AAV-IGF2. Nonetheless, some positive effects were also found for this AAV-IGF1 group compared to the sham-injected mice. Besides, according to several papers using AVV-GFP in the hippocampus, no additional effect in memory have been found when GFP is overexpressed in mice [5] [6] or in rats [7] hippocampi.

3) On page 6 of the manuscript, it is stated that "IGF2 treatment reverses ageing-related defects...". While this may certainly be the case, this claim cannot be made in light of the data presented, as no young control is provided for comparison. This is also claimed in the discussion on page 15

Thank you very much for this interesting comment. We have performed a new Morris water maze experiment comparing 7-month to 15-month-old wild type animals and the data are included in supplementary Figure 1. Comparing Figure 1 and supplementary Figure 1, you could observe that AAV-IGF2-treated mice behave similar to 7-month old wild type animals on probes on days 4 and 7, (reaching about 50% of time in the right quadrant) and, even better on probe on day 9 (63.4 % for IGF2 vs 49.29 % for 7-month-old). See page 6 in Results section.

4) Relating to the point above, one could claim that a 30% freezing is relatively low, but actually, if one compares the freezing levels of old sham-injected mice with that of the wt controls presented in subsequent figures (which are also old), it is quite remarkable that aged wt mice have an 80% freezing. Indeed, an 80% freezing is quite unlikely after a 0.3mA shock. Authors should comment on both these issues.

The data displaying in Figure 4A-B (showing the 80% of freezing for WT animals) correspond to a stronger paradigm of training, with 2 CS-US pairings, separated by a 10 s resting interval. The data in Figure 1D (showing a 30% of freezing for WT animals) correspond to a weaker paradigm of training, with only 1 shock, in order to detect an enhancement of memory in these aged WT animals that had a more preserved memory than the transgenic mice (see Methods, page 23).

5) On page 6 it is claimed that "although all the groups were able to remember the platform location...". This seems unlikely to be true for WT animals on the 4th day probe test. Please correct or provide statistical measure.

Thank you for the comment. We have deleted that sentence where it refers to all groups were able to remember the platform location in the probe of day 4. The referee is correct; the WT group spent about 25% of time in the right quadrant that is the chance level for swimming in any quadrant zone.

6) It would be helpful to get an explanation, why the MWM data from part 2 and part 5 show completely different baseline levels of escape latency, though the conditions for the wild-type control mice seem to be the same (assuming, that they are of the same age; unfortunately no information are given in part 2 about that)

Although the mean escape latency on day one for the WT animals (Figure 1F) is a little bit higher that the mean escape latency on day one for the WT animals in Figure 4C, the difference is not significant and is due to inter individual differences in the behaviour of the animals. However you can find a very similar behaviour pattern in the probes along the days, which represent more accurately memory retention. It should be taken into account that the Morris water maze is a task performed during a total of 12 consecutive days (3 days visible platform and 9 days hidden platform), and therefore, a single day should not be taken into account to draw general conclusions.

7) The finding that injection of the IGF2 AAV in the hippocampus reduces amyloid load in the cortex is quite fascinating. However at present there is no good explanation for this phenotype. The authors mention the spread of the virus from the hippocampus to the prefrontal cortex but as pointed out above (point 1) the characterization of the AVV- IGF2 is difficult and I skepctical if any conclusion can be drawn form the AAV-GFP image show in Fig 1A.

The reviewer is right as we could not confirm by IHC the over expression of AAV-IGF2 in the cortex. However, we confirmed, by PCR using primers against the viral IGF2 (different to that of endogenous IGF2), the fact that therapeutic viral IGF2 is expressed in the cortex and could reduce amyloid burden. As mentioned before, the viral vector we chose to deliver IGF2 into the brain has certain weaknesses; however, this implies that we can only differentiate the exogenous protein by RT-PCR (see explanation above).

Minor points:

1) On page 7, consider rephrasing the description of the culture data experiments. As it is now, it reads as if the Tg2567 cultures are to be treated with Ab as well.

The referee is right; thank you for the suggestion, we have modified the sentence.

2) Given that the line is available, authors should provide a measure of IGF2 protein levels in the brain of Tg2567 animals. Why go the trouble of looking in culture when it can be done in vivo and is more biologically relevant?

The referee is right; however, we found problems to detect endogenous IGF2 level in the mice hippocampus (see the comment above) using western blot and ELISA. With these techniques we could only detect IGF2 in the AAV-IGF2-injected animals. So, we tried the western blot in the neuronal culture, and since IGF2 is expressed in neurons, we succeed detecting IGF2 in neuronal culture by western blot. However, with the referee's comments, we have tried to work out the technical conditions to detect endogenous IGF2 in the mice hippocampus. See the answer above.

3) On page 11, authors claim that "only partially IGF1... reverses memory deficits". This is not really true for IGF1, as it is only seen in the fear conditioning and even there not fully. Please consider rephrasing to be more specific.

Thank you for the comment. We have considered you comment and we have deleted the sentence stating that IGF1 reverses memory deficits.

4) Sentences are sometimes really long and hard to read. There are some orthographical mistakes here and there (especially pertaining 3rd-person conjugation). Please revise.

Thank you for the comment. We have tried to detect and correct all the mistakes.

5) Some figures are wrongly referred to (i.e. figure 1C which is referred to as 1D, figure 7 in the discussion part). Also, some data are described in the text without any reference to the explaining figure, most likely because the figure was once mentioned 3 paragraphs before.

Thank you for the comment. We have tried to detect and correct all the mistakes.

Reviewer #3 (Comments to the Author):

- First, the lively debate of whether IGF1 signaling protects from AD (Carro et al., Nat Med. 2002 Dec;8(12):1390-7) or promotes toxicity (Freude et al., FASEB J. 2009 Oct;23(10):3315-24 and Cohen et al., Cell. 2009 Dec 11;139(6):1157-69.).

The referee is right according the lack of agreement between authors reporting the function of IGF1 in AD. We have included the references suggested by the referee in the manuscript [8, 9]. However, the main interest in our paper is mainly on IGF2 and not on IGF1. Importantly, Mellot et al. (2014) [10] study, and our manuscript are the first studies reporting a role for IGF2 in AD. Note that, although with very different approach, Mellot et al., [10] and our manuscript, confirmed that IGF2 ameliorates amyloid burden. Moreover, we went further and demonstrated that IGF2 rescues behavioural deficits, promote dendritic spine formation and restore normal hippocampal excitatory synaptic transmission in AD mice model. All together, we believe the manuscript have strong data to confirm a protective role for IGF2 in AD.

- Secondly, the authors tested the expression of IGF1 and IGF2 but did not provide evidences for possible functional outcomes. The claim that no IGF1 antibody is available (page 5, third paragraph) is not sufficient as they could test whether IGF-regulated transcription factors (e.g FoxO family members) change their cellular localization and whether known target genes are induced (for instance p27 or MnSOD).

As mentioned before, as functional outcome we demonstrated that the overexpression of IGF2 rescues behavioural deficits, promote dendritic spine formation and restore normal hippocampal excitatory synaptic transmission in AD mice model.

We confirmed the expression of IGF1 by RT-PCR, demonstrating that the viral construction targeted the hippocampus and that viral IGF1 is expressed in the targeted area. Unfortunately, due to the lack of a specific antibody, we could not fully complete the IGF1 expression study as we did for the IGF2. We thank the suggestion of the referee but the study of IGF1 target genes is out of our interest, as I mentioned before, the main interest in our paper is mainly IGF2 and not IGF1.

- The analysis of Aβ levels is also fall short of being sufficient. It is widely accepted that small Aβ oligomers, not fibrils are the most toxic species (Shankar et al., Nat Med. 2008 Aug;14(8):837-42 and Cohen et al., Cell. 2009 Dec 11;139(6):1157-69.). Thus, it is critical to employ size exclusion chromatography and ascertain the ration of monomers, oligomers and fibrils. Furthermore, the authors exclusively measured the amounts of Aβ 42 while the ratio of Aβ40 to Aβ42 has been shown to be critically important.

Thank you for the comment. As recommended, we have now assayed Aβ40 levels in prefrontal and parietotemporal cortex by ELISA kit (Invitrogen, Camarillo, CA) following the protocol according to the manufacturer's instructions. Our new data showed that Aβ40 decreased significantly in AAV-IGF2 compared to sham and AAV-IGF1-injected mice. The new data are included in Figure 6B.

We agree that it would be a valuable addition to study the ratio of monomers, oligomers and fibrils. We will go further and study deeper how amyloid pathology develops in Tg2576 mice injected with AAV-IGF2. However, in the present study we showed a decrease in amyloid burden in the AAV-IGF2-injected mice that could help the restoration of synaptic function. These results indicate that the toxic forms of amyloid are decreasing, since a functional positive outcome is evident in the behavioural and electrophysiology data in the treated animals.

- I also find the claims that the over-expression of IGF2 improves synaptic activity not appropriately tested as no direct measurement of synaptic activity is presented.

We mentioned that IGF2 improves synaptic transmission and not synaptic activity. Regarding this point, miniature EPSCs are a way to measure synaptic transmission. We rescue the frequency of the miniatures, not the amplitude and this might say that the synapse is not completely recovered after the 4 months treatment. Nevertheless miniature EPSCs is a synaptic and also circuit measurement (many synapses into a particular cell) that permits to look to basal transmission.

- The effect of IGF2 on cell viability should be tested in cultured cells by an adequate assay (for instance MTT assay).

The referee is right, we should measure weather the AAV-IGF2 (and AAV-IGF1 and AAV-Luc used as control) affect cell viability. Therefore, as suggested, we measured the lactate dehydrogenase (LDH) activity present in the culture medium of neurons treated with the different AAVs and untreated cells. LDH is a soluble cytosolic enzyme that is released into the culture medium following loss of membrane integrity resulting from either apoptosis or necrosis. LDH activity, therefore, can be used as an indicator of cytotoxicity. As depicted in the following figure, any of the treatments corresponding to Figure 7A in the manuscript, affects the viability of neurons.

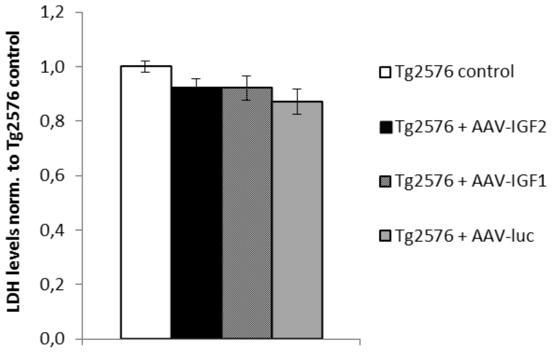

REFERENCES

[1] Ricobaraza A, Cuadrado-Tejedor M, Marco S, Perez-Otano I, Garcia-Osta A (2012) Phenylbutyrate rescues dendritic spine loss associated with memory deficits in a mouse model of Alzheimer disease. *Hippocampus*;22:1040-1050

[2] Dong H, Murphy KM, Meng L, Montalvo-Ortiz J, Zeng Z, Kolber BJ et al. (2012) Corticotrophin releasing factor accelerates neuropathology and cognitive decline in a mouse model of Alzheimer's disease. *J Alzheimers Dis*;28:579-592

[3] Perez-Cruz C, Nolte MW, van Gaalen MM, Rustay NR, Termont A, Tanghe A et al. (2011) Reduced spine density in specific regions of CA1 pyramidal neurons in two transgenic mouse models of Alzheimer's disease. *J Neurosci*;31:3926-3934

[4] Pietrzkowski Z, Wernicke D, Porcu P, Jameson BA, Baserga R (1992) Inhibition of cellular proliferation by peptide analogues of insulin-like growth factor 1. *Cancer Res*;52:6447-6451

[5] Kiyota T, Ingraham KL, Jacobsen MT, Xiong H, Ikezu T (2011) FGF2 gene transfer restores hippocampal functions in mouse models of Alzheimer's disease and has therapeutic implications for neurocognitive disorders. *Proc Natl Acad Sci U S A*;108:E1339-1348

[6] Hudry E, Van Dam D, Kulik W, De Deyn PP, Stet FS, Ahouansou O et al. (2010) Adeno-associated virus gene therapy with cholesterol 24-hydroxylase reduces the amyloid pathology before or after the onset of amyloid plaques in mouse models of Alzheimer's disease. *Mol Ther*;18:44-53

[7] Poulsen DJ, Standing D, Bullshields K, Spencer K, Micevych PE, Babcock AM (2007) Overexpression of hippocampal Ca2+/calmodulin-dependent protein kinase II improves spatial memory. *J Neurosci Res*;85:735-739

[8] Cohen E, Paulsson JF, Blinder P, Burstyn-Cohen T, Du D, Estepa G et al. (2009) Reduced IGF-1 signaling delays age-associated proteotoxicity in mice. *Cell*;139:1157-1169

[9] Freude S, Hettich MM, Schumann C, Stohr O, Koch L, Kohler C et al. (2009) Neuronal IGF-1 resistance reduces Abeta accumulation and protects against premature death in a model of Alzheimer's disease. *Faseb J*;23:3315-3324

[10] Mellott TJ, Pender SM, Burke RM, Langley EA, Blusztajn JK (2014) IGF2 Ameliorates Amyloidosis, Increases Cholinergic Marker Expression and Raises BMP9 and Neurotrophin Levels in the Hippocampus of the APPswePS1dE9 Alzheimer's Disease Model Mice. *PLoS One*;Apr 14

2nd Editorial Decision 30 June 2014

Thank you for the submission of your revised manuscript to EMBO Molecular Medicine. We have now received the enclosed reports from the referees that were asked to re-assess it. As you will see the reviewers are now globally supportive and I am pleased to inform you that we will be able to accept your manuscript pending the following final amendments:

Please address the remaining issues highlighted by the referees. Please provide the figure asked by referee 1 as SI figure and relabel the rest of the SI figures appropriately if needed. Do not forget to call the SI figures accordingly within the main text. Please address referees 2's concerns in writing and address referee 3's last issue as best as you can; do include the (Mellot et al 2014) citation, discussing it accordingly.

Please submit your revised manuscript within two weeks.

I look forward to reading a new revised version of your manuscript as soon as possible.

***** Reviewer's comments *****

Referee #1 (Remarks):

The authors have addressed most of my comments in the revised version of the manuscript. There is one comment remaining, which I feel needs to be addressed before giving the final ok for publication.
Concerning the low resolution image of the virus injection: I would tend to insist that such image is indeed necessary to include. Of course, soma and neuropils cannot be distinguished, but that is not the point. The point is that by including a low resolution picture (as the authors have done in the rebuttal letter) the reader knows that the injection really was targeting the hippocampus. Perhaps an overlay between DAPI and GFP will provide a better picture of the entire structure of the hippocampus. This should be included in the supplementary material
Concerning the quadrants in the morris water maze: This is unfortunate, and should be improved in future studies.

Referee #2 (Remarks):

I now read the revised version of the manuscript presented by Pascual-Lucas et al. and only two comments remain. The authors could address by rewording the manuscript.

Regarding response to Major point 1:
The statement that IGF1 or IGF2 would not fit into an AAV is wrong in my view. The capacity of the AAV is about 3500 bp. IGF1 and IGF2 cDNAs are both below 600 bp so that generation of a GFP or myc tagged version would not have been too problematic. Also a number of studies have used IGF1 and IGF2 ABs for immuno-staining. However, I am able to accept this explanation.

What I find still very difficult is the experimental design using sham operated mice as control. The authors changed their interpretation now stating that IGF1 injected mice serve as control and cite studies showing that AAV mediated expression of GFP does not alter behavior in mice. However, non of the cited studies compared an AAV group against a sham group as far as I see. It would indeed be an interesting experiment to see if the expression of an AAV without the gene of interest would change behavior in comparison to a sham group.
However, the authors could also address this issue in greater detail in the text. Moreover, taking into account that similar data are now available, this study further indicates the important role of IGF2 in Alzheimer's disease.
Another way to strengthen the authors approach would also be the finding that IGF2 is now down-regulated during aging. Maybe IGF1 is not and this is why IFG1 has only a mild effect when overexpressed?

Regarding response to Major point 6:
The authors should explain this issue in the manuscript

Referee #3 (Comments on Novelty/Model System):

The model systems are adequate.
The findings are interesting and the study addresses an important question, however, a similar study that has been published recently should be brought to the reader's attention (Mellott et al., PLoS One 2014).

Referee #3 (Remarks):

he authors have improved the manuscript but need to address the following issues. First, they did not cite the recent PLoS One paper (Mellot et al., 2014) which describes similar findings and thus, should be brought to the reader's attention. The article appears at the rebuttal letter but absent from the manuscript. In addition, they did not address the issue of A-beta assemblies (oligomers versus fibrils) but make claims regarding amyloid burden. In my opinion, in the light of the current knowledge in the field showing that oligomers as the major toxic agents it is required to analyze the relative oligomers load in the different mouse groups.

2nd Revision - authors' response 11 July 2014

Response to Reviewers

EMBO Molecular Medicine, Manuscript ID EMM-2014-04228-T: "Insulin-like growth factor 2 reverses memory and synaptic deficits in APP transgenic mice"

We thank the editor and reviewers for their careful reading and helpful comments. We have revised the manuscript in response to some remaining issues highlighted by the referees and the editor.

Editor (Remarks):

We have included a new figure as Supplementary Figure 1 as suggested by the referee #1, we have tried to include the comments by referee #2 (in last paragraph of the introduction and in results section, page 6). We have also included Mellot et al. 2014 citation as suggested by the referee #3, discussing it accordingly (discussion section, page 18).

All the remaining issues highlighted by the referees are detailed in the point-by-point responses below.

Referee #1 (Remarks):

The authors have addressed most of my comments in the revised version of the manuscript. There is one comment remaining, which I feel needs to be addressed before giving the final ok for publication.

Concerning the low resolution image of the virus injection: I would tend to insist that such image is indeed necessary to include. Of course, soma and neuropils cannot be distinguished, but that is not the point. The point is that by including a low resolution picture (as the authors have done in the rebuttal letter) the reader knows that the injection really was targeting the hippocampus. Perhaps an overlay between DAPI and GFP will provide a better picture of the entire structure of the hippocampus. This should be included in the supplementary material.

We agree that in the low resolution picture you can see that the hippocampus is targeted. The figure is included in supplementary Figure 1.

Concerning the quadrants in the morris water maze: This is unfortunate, and should be improved in future studies.

Thank you for your suggestion, this issue will be improved in future studies.

Referee #2 (Remarks):

Regarding response to Major point 1:

The statement that IGF1 or IGF2 would not fit into an AAV is wrong in my view. The capacity of the AAV is about 3500 bp. IGF1 and IGF2 cDNAs are both below 600 bp so that generation of a GFP or myc tagged version would not have been too problematic. Also a number of studies have used IGF1 and IGF2 ABs for immuno-staining. However, I am able to accept this explanation.

Thank you very much for your understanding.

What I find still very difficult is the experimental design using sham operated mice as control. The authors changed their interpretation now stating that IGF1 injected mice serve as control and cite studies showing that AAV mediated expression of GFP does not alter behavior in mice. However, non of the cited studies compared an AAV group against a sham group as far as I see. It would indeed be an interesting experiment to see if the expression of an AAV without the gene of interest would change behavior in comparison to a sham group.

However, the authors could also address this issue in greater detail in the text. Moreover, taking into account that similar data are now available, this study further indicates the important role of IGF2 in Alzheimer's disease.

Another way to strengthen the authors approach would also be the finding that IGF2 is now down-regulated during aging. Maybe IGF1 is not and this is why IFG1 has only a mild effect when overexpressed?

We agree that to have an AAV without any gene would be the best control and it is a highly interesting point. Nevertheless, we did not change the interpretation stating AAV-IGF1 injected mice serve as control, since we decided to use IGF1 assuming that it would not have any effect in memory (see the paper by Chen et al., 2011, Nature). Therefore, we thought that the best control was to inject an AAV producing an insulin growth factor with no function in memory. Despite everything, the referee is right and the best control should be a group injected with AAV without any gene.

We do not have studied IGF1 expression levels in aging or in pathological conditions, however, it is possible that IGF1 levels is less affected than IGF2 and that´s why the effect after IGF1 over expression is milder.

We have included these statements in the manuscript.

Regarding response to Major point 6:

The authors should explain this issue in the manuscript

We do not believe that this is an important point to be commented in the manuscript. As we mentioned in the rebuttal letter, the differences are small and can be due to inter individual differences. Moreover, the strains of the animals are different and also could contribute to some variation in the escape latency.

Referee #3 (Comments on Novelty/Model System):

The model systems are adequate.

The findings are interesting and the study addresses an important question, however, a similar study that has been published recently should be brought to the reader's attention (Mellott et al., PLoS One 2014).

Thank you for the suggestion. We have included and commented the citation by Mellott et al., PLoS One, 2014. However, we disagree with the referee in the sense that the studies are very different. Besides the different system used by Mellott et al., (recombinant protein and 6-month-old APP/PS1 mice) they focused their attention in the amyloid pathology study, whereas our manuscript is beyond the amyloid pathology in aged Tg2576 mice. Furthermore, the results obtained are different since they don´t see any difference in total $A\beta_{42}$ and $A\beta_{40}$ levels and they observed a reduction in amyloid deposits. We found a decrease in total $A\beta_{42}$ and $A\beta_{40}$ levels using ELISA analysis.

Referee #3 (Remarks):

The authors have improved the manuscript but need to address the following issues. First, they did not cite the recent PLoS One paper (Mellot et al., 2014) which describes similar findings and thus, should be brought to the reader's attention. The article appears at the rebuttal letter but absent from the manuscript. In addition, they did not address the issue of A-beta assemblies (oligomers versus fibrils) but make claims regarding amyloid burden. In my opinion, in the light of the current knowledge in the field showing that oligomers as the major toxic agents it is required to analyze the relative oligomers load in the different mouse groups.

Thank you for the suggestion. We are working in this subject now, however, it would take time and we think the actual data are robust enough for publication. To provide such data would be a project by itself and would delay our message loosing more novelty.
