# Supplementary figures and images for: Insulin-like growth factor 2 reverses memory and synaptic deficits in APP transgenic mice
Source: EMBO Mol Med. 2014 Aug 7;6(10):1246–62. doi: 10.15252/emmm.201404228 (PMC4287930; doi:10.15252/emmm.201404228)

Figure 2

A

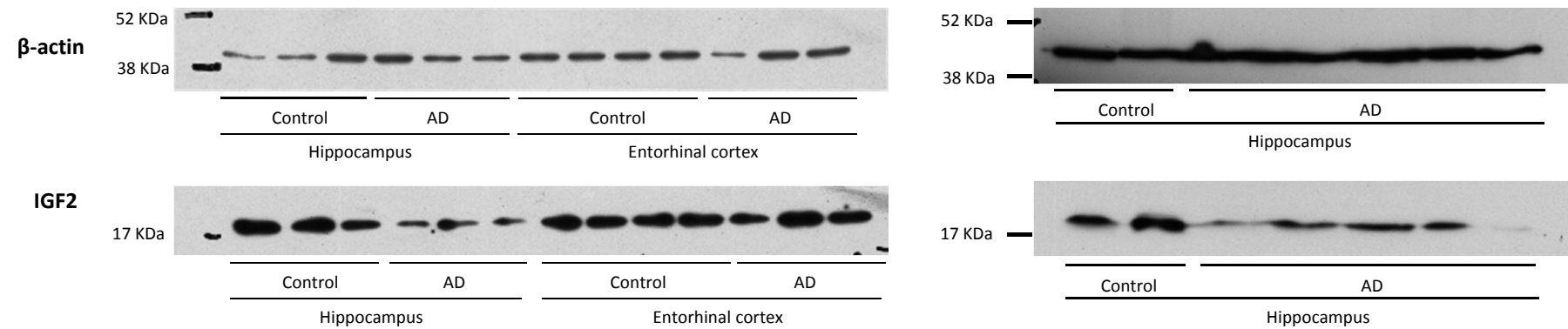

B

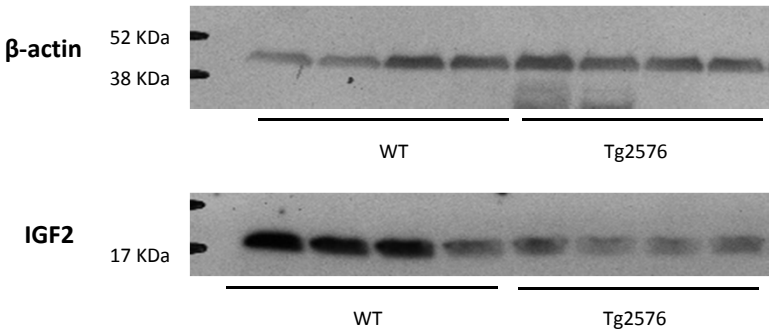

C

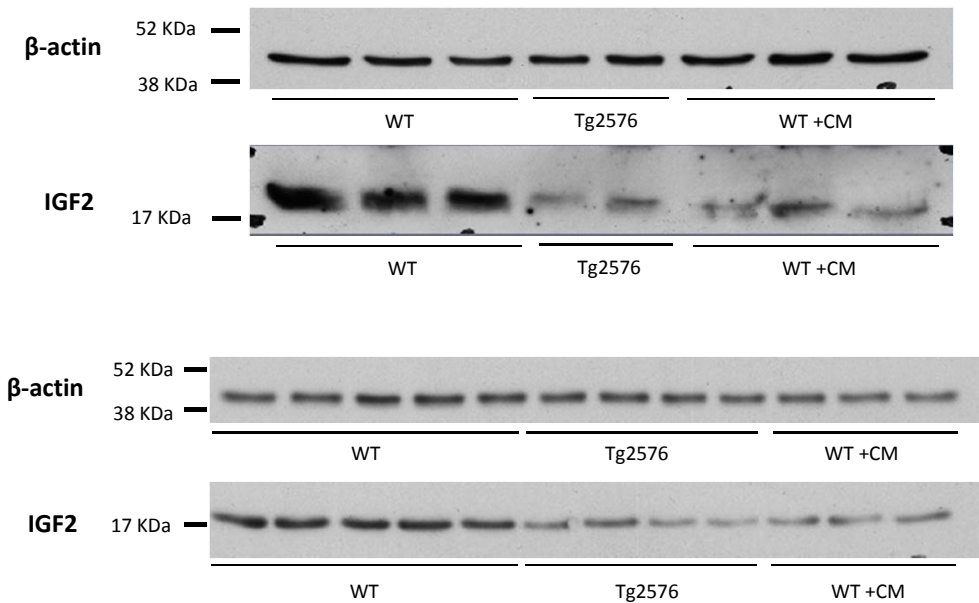

Supplement: Supplementary file 3 [file emmm0006-1246-sd3.pdf]

Figure 3

A

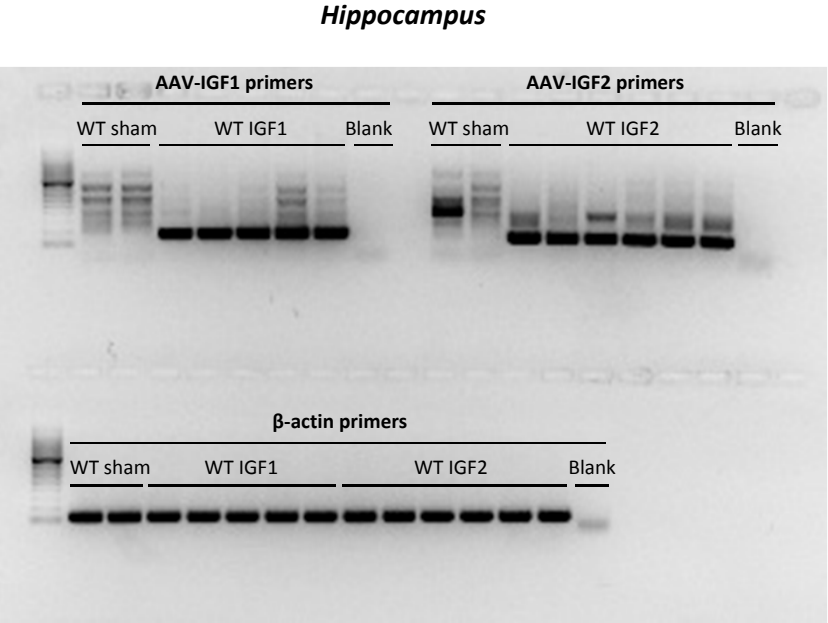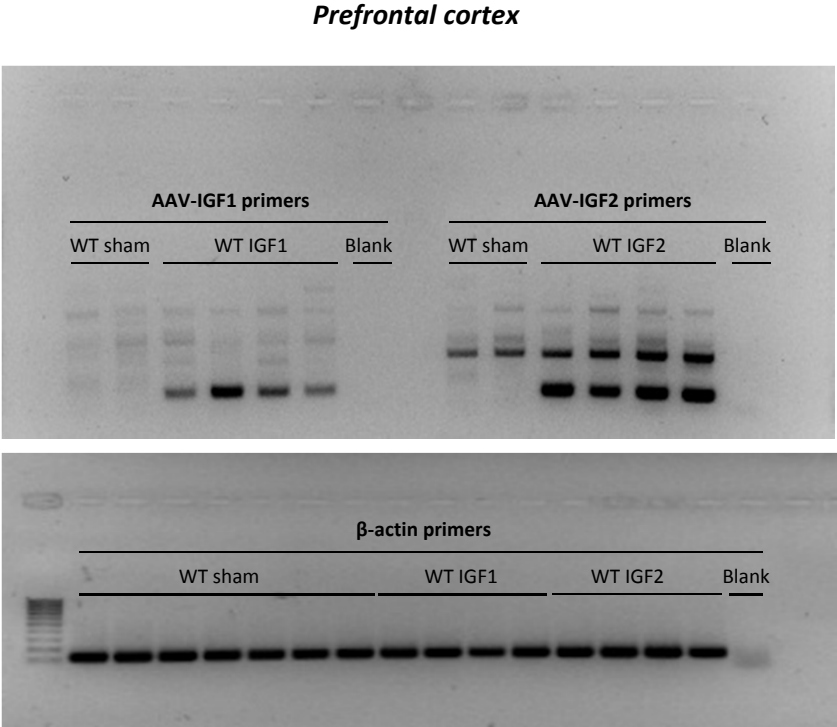

C

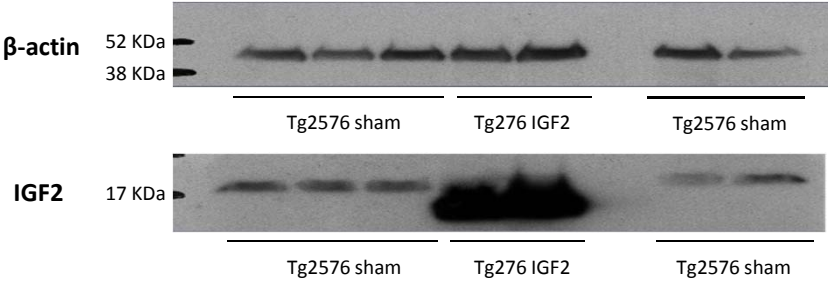

Supplement: Supplementary file 4 [file emmm0006-1246-sd4.pdf]
